# Structural basis of Integrator-dependent RNA polymerase II termination

**DOI:** 10.1038/s41586-024-07269-4

**Published:** 2024-04-03

**Authors:** Isaac Fianu, Moritz Ochmann, James L. Walshe, Olexandr Dybkov, Joseph Neos Cruz, Henning Urlaub, Patrick Cramer

**Affiliations:** 1 Department of Molecular Biology, Max Planck Institute for Multidisciplinary Sciences, Göttingen, Germany; 2 Bioanalytical Mass Spectrometry, Max Planck Institute for Multidisciplinary Sciences, Göttingen, Germany; 3 Institute of Clinical Chemistry, Bioanalytics Group, University Medical Center Göttingen, Göttingen, Germany; 4 Cluster of Excellence ‘Multiscale Bioimaging: from Molecular Machines to Networks of Excitable Cells’ (MBExC), University of Göttingen, Göttingen, Germany

**Keywords:** Transcription, Cryoelectron microscopy

## Abstract

The Integrator complex can terminate RNA polymerase II (Pol II) in the promoter-proximal region of genes. Previous work has shed light on how Integrator binds to the paused elongation complex consisting of Pol II, the DRB sensitivity-inducing factor (DSIF) and the negative elongation factor (NELF) and how it cleaves the nascent RNA transcript^1^, but has not explained how Integrator removes Pol II from the DNA template. Here we present three cryo-electron microscopy structures of the complete Integrator–PP2A complex in different functional states. The structure of the pre-termination complex reveals a previously unresolved, scorpion-tail-shaped INTS10–INTS13–INTS14–INTS15 module that may use its ‘sting’ to open the DSIF DNA clamp and facilitate termination. The structure of the post-termination complex shows that the previously unresolved subunit INTS3 and associated sensor of single-stranded DNA complex (SOSS) factors prevent Pol II rebinding to Integrator after termination. The structure of the free Integrator–PP2A complex in an inactive closed conformation^2^ reveals that INTS6 blocks the PP2A phosphatase active site. These results lead to a model for how Integrator terminates Pol II transcription in three steps that involve major rearrangements.

## Abstract (web summary)

Cryo-electron microscopy structures of the human Integrator complex in three different functional states shed light on how Integrator terminates RNA polymerase II (Pol II) transcription by disengaging Pol II from the DNA template.

## Main

Transcription by RNA polymerase II (Pol II) controls organismal development and cellular responses to environmental cues^3^. In metazoans, Pol II transcription is regulated during promoter-proximal pausing^4^ when the elongation factors DSIF and NELF assemble with Pol II to form the paused elongation complex (PEC)^5^. Pausing often occurs close to a well-positioned +1 nucleosome located downstream of the pause site^6,7^. Paused Pol II proceeds into productive elongation after Pol II, DSIF and NELF are phosphorylated by the P-TEFb kinase complex, leading to the dissociation of NELF and the binding of SPT6 and the PAF1 complex (PAF1c)^4,8^. Alternatively, the PEC can undergo premature termination (also called attenuation) after the binding of the Integrator complex and PP2A^1,9–15^. Integrator not only targets paused Pol II during the transcription of protein-coding genes, but is also involved in terminating the synthesis of noncoding Pol II transcripts, including enhancer RNAs^16^, small nuclear RNAs^17^, telomerase RNA^18^ and long noncoding RNAs^19^. Indeed, Integrator is a genome-wide regulator of Pol II transcription^20,21^.

Integrator is an approximately 1.5-MDa complex that was at first thought to consist of 14 subunits called INTS1–INTS14 (refs. ^17,22^). The protein C7orf26 was then found to be an additional subunit of Integrator, and was named INTS15 (ref. ^23^). Structural analysis of the Integrator–PP2A complex in the absence of INTS15 visualized nine Integrator subunits and the intimately associated PP2A complex^1,2^. These structures show that the Integrator–PP2A complex is in a closed and inactive conformation that opens after binding to the PEC to adopt an enzymatically active conformation^1,2^. Subunits INTS4, INTS9 and INTS11 constitute the Integrator cleavage module^24^, which contains endonuclease activity^1,2^ and resembles the cleavage module of the cleavage and polyadenylation specific factor (CPSF) that is required for 3′ processing of mRNA transcripts^25–27^. The Integrator cleavage module docks at the RNA exit tunnel of Pol II such that the exiting nascent RNA would proceed directly into the endonuclease active site for cleavage when Integrator binds to the PEC (ref. ^1^). The subunits INTS1, INTS2 and INTS5–INTS8 form the core of Integrator, in which INTS2, INTS5, INTS6 and INTS8 bind to and position PP2A to dephosphorylate the C-terminal domain (CTD) of Pol II subunit RPB1 (refs. ^1,2,15,28^).

Previous structures of Integrator did not reveal INTS3, INTS10, INTS12, INTS13 and INTS14, owing to flexibility^1,2^. The C-terminal region of INTS3 binds to the flexible C-terminal region of INTS6 (refs. ^29,30^) in the Integrator complex, whereas the N-terminal region binds to NABP1 or NABP2 (human SSB1 or SSB2) and INIP in the SOSS complex, which is involved in the DNA damage response^31^. NABP2 and INIP co-localize with Integrator at Pol II pause sites and physically interact with the Integrator complex^13,32^, which suggests that these factors have a role in Integrator-dependent Pol II termination. The single-stranded-DNA-binding activity of NABP2 was recently proposed to recruit an Integrator–PP2A–SOSS complex to paused Pol II for premature termination to ensure genome stability^32^. The flexible subunits INTS10, INTS13 and INTS14 form the INTS10–INTS13–INTS14 module that was suggested to bind to nucleic acids^33,34^. This module interacts with the new subunit INTS15 to form a tetrameric INTS10–INTS13–INTS14–INTS15 subcomplex^23,35^. INTS12 binds to the flexible N terminus of INTS1, has a predicted plant homeodomain and may bind to DNA and histone tails^1,17^. The roles of these flexible Integrator subunits and the SOSS factors in Integrator function are unclear at the molecular level.

Integrator-dependent Pol II termination is thought to occur in three steps. First, Integrator binds to the PEC and the phosphatase activity of the Integrator-associated PP2A dephosphorylates the Pol II CTD and the C-terminal region of the DSIF subunit SPT5 (refs. ^2,15,28^). Consequently, PP2A opposes the kinase activity of P-TEFb and prevents the association of the positive elongation factors SPT6 and PAF1c. Integrator also sterically occludes the binding of these factors to the Pol II surface and therefore commits the PEC to a termination pathway after binding^1^. Second, the endonuclease activity in INTS11 cleaves the exiting nascent RNA, generating an uncapped RNA 5′ end^1,9–11^. The third and final step requires the unravelling of the DNA–RNA hybrid and the release of the DNA that is locked in the Pol II cleft by two clamps—the Pol II clamp^36^ and the DSIF DNA clamp^37^. How this final step of Pol II termination is achieved by Integrator is unknown. Here we report three cryo-electron microscopy (cryo-EM) structures that show previously unresolved Integrator subunits and the Integrator-associated SOSS factors. Together, these structures provide insights into how Integrator terminates Pol II, leading to a dynamic model for Integrator action.

## Cryo-EM analysis

**Extended Data Fig. 1:**
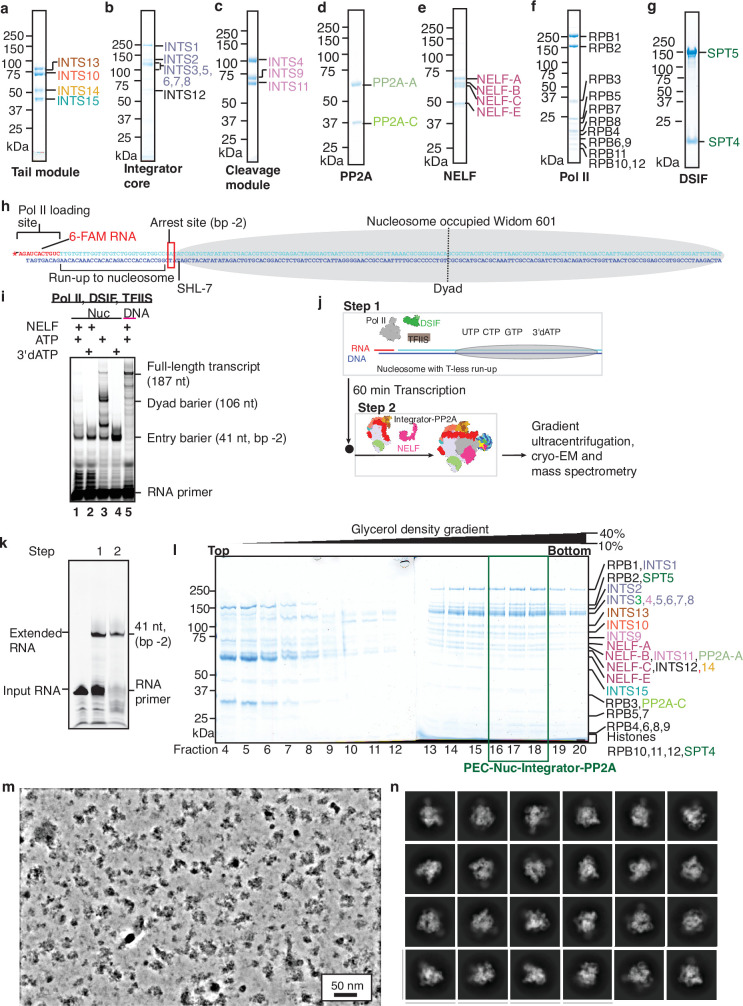
Quality of proteins, reconstitution of the pre-termination complex and cryo-EM images. Representative Coomassie-stained SDS–PAGE gels for: **a**, Integrator tail modules; **b**, Integrator core; **c**, Cleavage module; **d**, PP2A; **e**, NELF; **f**, Pol II and **g**, DSIF. **h**, Cartoon showing the DNA sequence used for nucleosome reconstitution and complex formation. The Cy5-labelled RNA used for loading Pol II and the arrest site at base pair −2 before the nucleosome bound Widom 601 are shown. **i**, RNA extension assay performed using the nucleosome and DNA substrates described in **h**. The assay was performed with Pol II, DSIF and TFIIS with and without NELF. To stall Pol II at the bp −2 we used 3′-dATP instead of ATP in the assay. **j**, Reconstitution of the pre-termination complex for cryo-EM. **k**, Denaturing PAGE showing the RNA product after steps 1 and 2 described in **j**. **l**, SDS–PAGE analysis of fractions taken from glycerol-gradient purification of the pre-termination complex. Proteins were identified by mass spectrometry. **m**, Representative micrograph of the pre-termination complex. The micrograph was denoised using Warp. A 50 nm scale bar is provided. **n**, Two-dimensional class averages of the selected good particles.

To investigate how the Integrator–PP2A complex terminates Pol II, we studied how the recently described subunit INTS15 (refs. ^23,35,38^) and previously unresolved subunits INTS3, INTS10, INTS12, INTS13 and INTS14 might facilitate the removal of Pol II from the template DNA after RNA cleavage. We prepared the recombinant human Integrator–PP2A complex as described previously^1^. In brief, we mixed four purified subcomplexes; namely, a tetrameric subcomplex containing INTS10, INTS13, INTS14 and INTS15, hereafter called the tail module (Extended Data Fig. 1a), the eight-subunit Integrator core (Extended Data Fig. 1b), the cleavage module (Extended Data Fig. 1c) and PP2A (Extended Data Fig. 1d)^1^. Human NELF (Extended Data Fig. 1e), mammalian Pol II (Extended Data Fig. 1f) and human DSIF (Extended Data Fig. 1g) were also purified (Methods). We designed a DNA template that allows Pol II transcription into a nucleosome (Methods and Extended Data Fig. 1h). We performed an in vitro RNA extension assay on a nucleosomal substrate prepared with this DNA template using Pol II, DSIF, NELF and TFIIS. The assay showed that NELF induces Pol II pausing with a predominant effect at base pair –2 before super-helical location –7 of the nucleosome. In the absence of NELF, the Pol II–DSIF complex transcribed beyond base pair −2 and stalls at the dyad barrier of the nucleosome (Extended Data Fig. 1h,i).

For cryo-EM analysis, we performed in vitro RNA extension on the nucleosomal substrate using Pol II, DSIF and TFIIS (Methods) in the presence of 3′-dATP to stall Pol II at base pair –2 (Pol II–DSIF–Nuc complex; Nuc indicates nucleosome). We then added NELF and the preformed Integrator–PP2A to the stalled Pol II–DSIF–Nuc complex and purified the resulting complex by glycerol-gradient ultracentrifugation (Methods and Extended Data Fig. 1j,k). SDS–PAGE and mass-spectrometry analyses showed that the Integrator–PP2A complex and NELF bound to the stalled Pol II–DSIF–Nuc complex to form a PEC–Nuc–Integrator–PP2A complex, hereafter called the pre-termination complex (Extended Data Fig. 1l). We subjected the complex to single-particle cryo-EM and cross-linking mass spectrometry (XL-MS) analyses.

**Extended Data Fig. 2:**
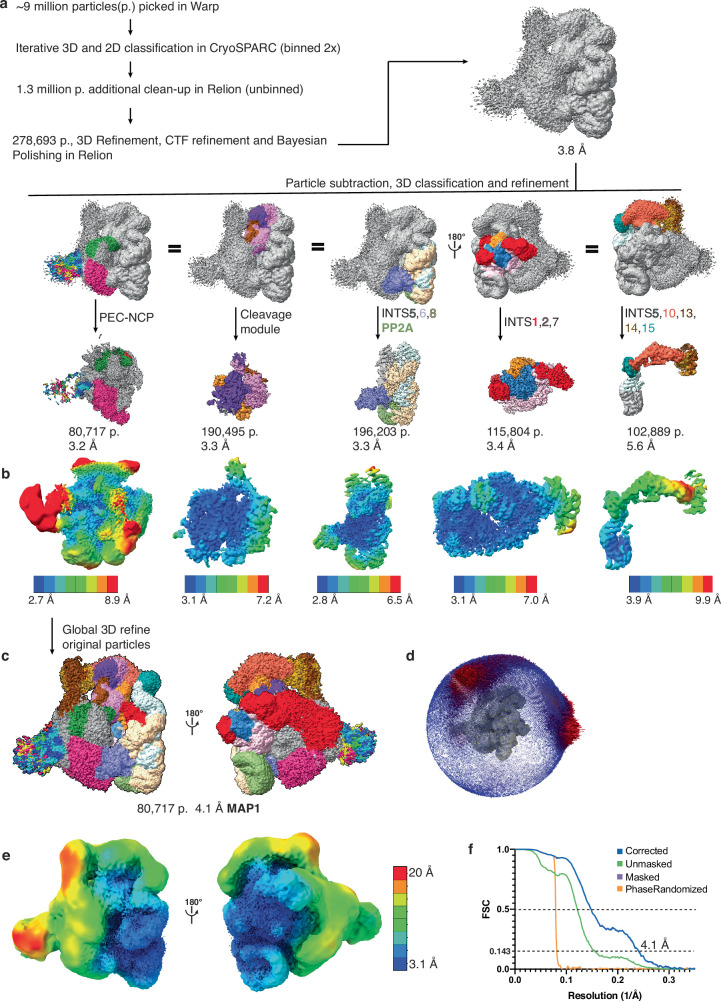
Cryo-EM data processing and resolution of the cryo-EM maps of the pre-termination complex. **a**, Flow chart illustrating key data-processing steps. **b**, Local resolution of focus refinement maps. Keys showing resolution values of the various coloured regions are provided. **c**, Overall cryo-EM reconstruction of the pre-termination complex. **d**, Angular distribution plot for the overall reconstruction. **e**, Overall reconstruction coloured according to local resolution. **f**, Fourier shell correlation (FSC) between the two half maps obtained from independent halves of the data. The gold-standard FSC at 0.143 is indicated.

**Extended Data Fig. 3:**
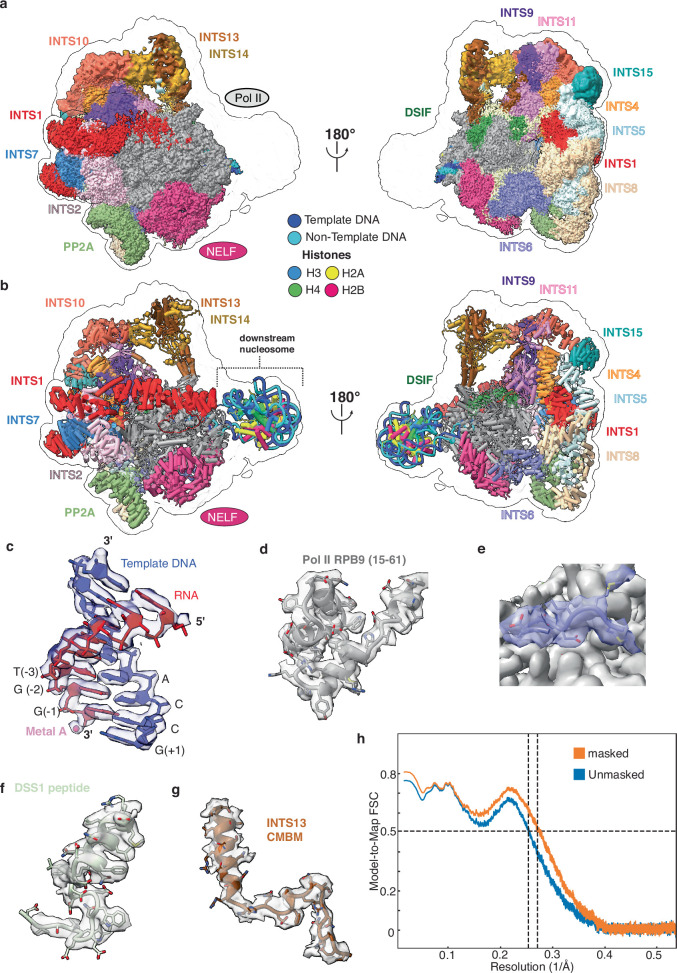
Fit of model to the cryo-EM map. **a**, Two views of the focused refined maps used for modelling fitted into a low-pass-filtered version of the overall reconstruction that is shown by the black outline. **b**, Two views of the overall structure fitted into the low-pass-filtered version of the overall reconstruction. **c**, Cryo-EM density for the DNA–RNA hybrid bound within the Pol II cleft. **d**, Cryo-EM density for Pol II RPB9(15-61). **e**, Cryo-EM density for INTS6 inhibitory loop at the PP2A-C active site **f**, Cryo-EM density for DSS1 **g**, Cryo-EM density for INTS13 CMBM. **h**, Model-to-map FSC plot.

**Extended Data Fig. 4:**
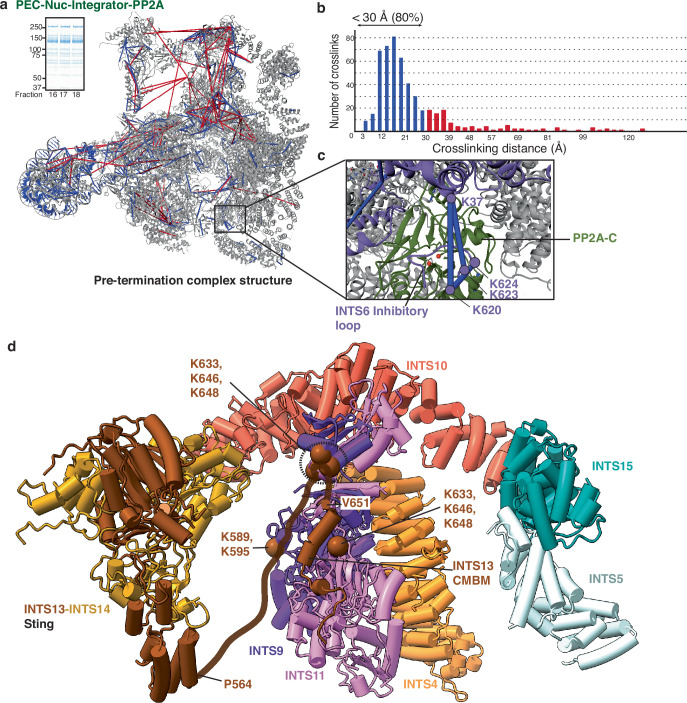
XL-MS analysis of the pre-termination complex. **a**, SDS–PAGE of the peak fractions of the pre-termination complex that was cross-linked with BS3. Mapping of the BS3 cross-links onto the pre-termination complex structure using Xlink Analyzer^71^. For clarity, only cross-links with >10 cross-linked peptide spectrum matches were included. **b**, Histogram showing distribution of distances between Cα pairs of cross-linked Lys residues in the pre-termination complex structure. **c**, Close-up view showing cross-links from INTS6 K37 to INTS6 K620 and K623 that are located just before the INTS6 inhibitory loop. **d**, Cartoon representation of the tail module together with the cleavage module and INTS5. Cross-link sites of the INTS13 flexible linker (residues 565–650) that connects the INTS13 sting to the INTS13 CMBM are shown as spheres and labelled with the INTS13 cross-linked residues. A putative path of the flexible linker is shown by the brown line.

During cryo-EM image processing, we found a subset of particles containing cryo-EM density for the pre-termination complex (Extended Data Fig. 1m,n). We obtained a cryo-EM reconstruction at an overall resolution of 4.1 Å (Extended Data Figs. 2 and 3, Extended Data Table 1 and Supplementary Video 1). Compared with our previous structure^1^, we observed additional cryo-EM density for the nucleosome and Integrator subunits. The density for the nucleosome was weak and lacked any additional unmodelled cryo-EM density, suggesting that Integrator does not bind stably to it (Extended Data Figs. 2 and 3b). This is consistent with the lack of cross-links between Integrator and the histones (Extended Data Fig. 4a and Supplementary Table 1). However, we observed a large additional density that emerges from the N terminus of the Integrator subunit INTS5 and runs around the Integrator cleavage module towards the upstream DNA. We used focus classification to improve this density and assigned it to the tail module (INTS10–INTS13–INTS14–INTS15) (Extended Data Fig. 2).

## Structure of the pre-termination complex

**Fig. 1:**
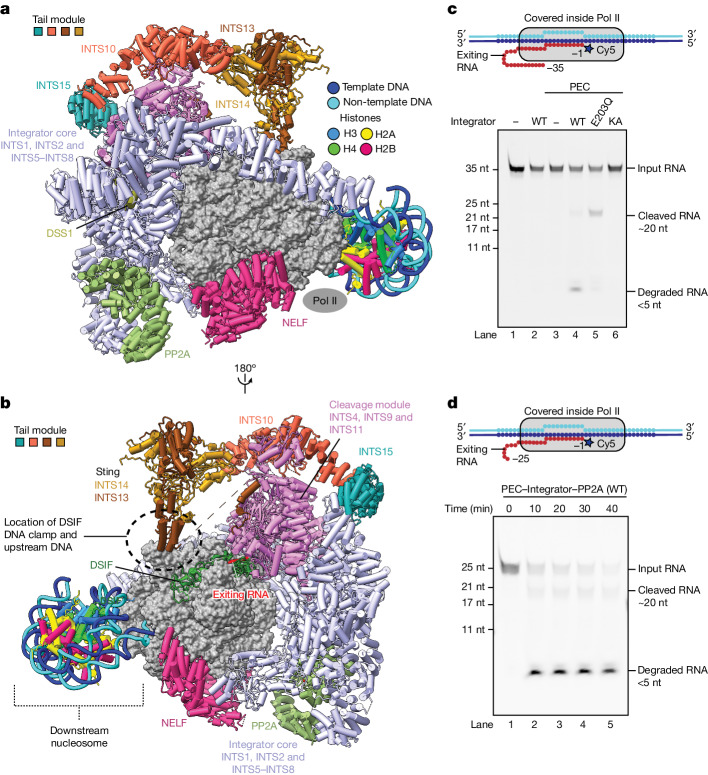
Cryo-EM structure of the pre-termination complex and Integrator activity. **a**, Overall structure of the complex. **b**, Alternative view of the complex. Dashed black circle indicates the canonical location of the DSIF DNA clamp and upstream DNA that is occupied by the sting of the tail module. **c**, Integrator cleaves and degrades PEC-bound RNA. Top, the nucleic-acid scaffold used in the assay. Note the Cy5 fluorescent label at the RNA 3′ end. Bottom, representative denaturing gel of an assay performed with Integrator containing wild-type INTS11 (WT), the INTS11(E203Q) mutant (E203Q) or the INTS11(D72K/H73A) mutant (KA). **d**, Time-dependent degradation of PEC-bound RNA by wild-type Integrator. The nucleic-acid scaffold used contains a mismatch bubble for a more efficient PEC assembly. The assays in **c**,**d** were performed three times.

To build an atomic model for the pre-termination complex, we docked the previous structure of PEC–Integrator–PP2A (Protein Data Bank (PDB) ID: 7PKS) into the overall cryo-EM density map and adjusted it against focused refinement maps. The DNA–RNA hybrid bound within the Pol II cleft was modelled de novo using a 3.2-Å focused refinement map around Pol II. This allowed us to determine the sequence register for the nucleic acids and to rigid body dock a model for the nucleosome (PDB ID: 7OHC). To model the tail module, we used AlphaFold2 models^39^ for INTS10 and INTS15 and the available crystal structure for the INTS13–INTS14 dimer (PDB ID: 6SN1). We fitted these models into the focused refined maps of these subunits and adjusted them using ISOLDE^40^. The interface between INTS10 and INTS14 was initially generated using ColabFold^41^ and agrees with our cryo-EM density map and previous biochemical data^34^. The complete structure was refined in real space using PHENIX^42^. The resulting model showed good stereochemistry (Fig. 1a,b, Extended Data Fig. 3, Extended Data Table 1 and Supplementary Video 1) and was further confirmed by the XL-MS data (Extended Data Fig. 4a and Supplementary Table 1).

**Fig. 2:**
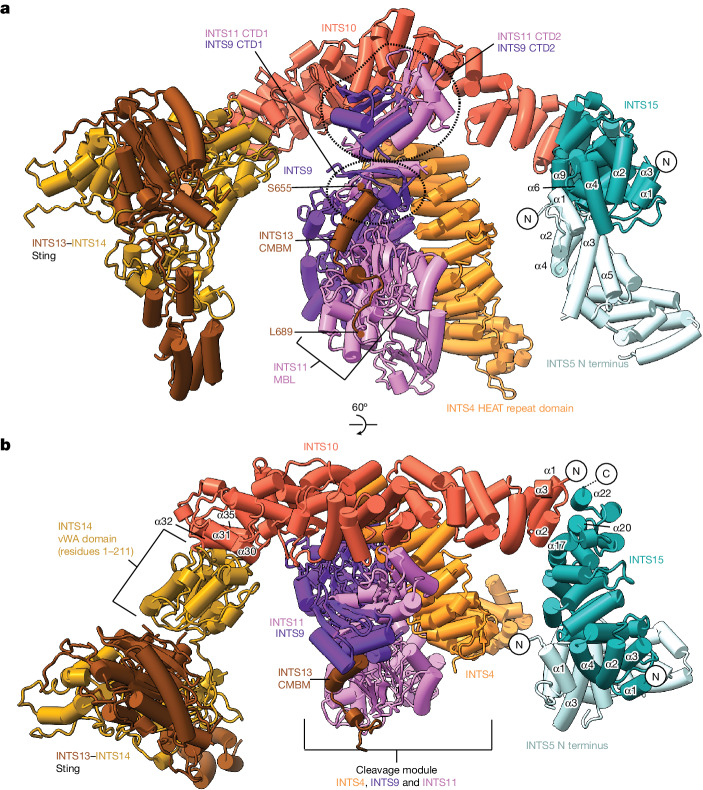
Architecture of the Integrator tail module. **a**, Cartoon rendering of the tail module together with the Integrator cleavage module and the INTS5 N-terminal region. **b**, Alternative view. MBL, metallo-β-lactamase domain.

The overall structure resembles our previous structure^1^ (Fig. 1a,b) except for the following. The complex adopts the post-translocated state (Extended Data Fig. 3c), and the DNA–RNA hybrid is not tilted as observed in the PEC (refs. ^1,5^). The observed Pol II active site is located four nucleotides (nt) upstream of our designated stall position, showing that Pol II backtracked after stalling and that the backtracked RNA was cleaved by TFIIS. The Pol II active site is located around 80 nt from the nucleosomal dyad, consistent with the first nucleosomal barrier encountered by Pol II during transcription^7,43^. The structure also reveals the previously unresolved Integrator tail module (Figs. 1 and  2). Unexpectedly, during cryo-EM data analysis, we found cryo-EM density for co-purified insect-cell DSS1, a protein known to interact with Integrator^17^, bound to INTS7 (Fig. 1a, Extended Data Fig. 3f and Supplementary Fig. 1). The cryo-EM density for DSS1 was present in previous Integrator structures but could not be assigned^1,2,44^. DSS1 binds to Integrator, the proteosome, the COP9 signalosome, TREX2 and BRCA2, and serves a scaffolding function in these complexes^45^. Only the subunits INTS3 and INTS12 remain unresolved in the structure of the pre-termination complex.

## Structure of the Integrator tail module

The overall structure of the tail module is shaped like a scorpion’s tail, with INTS15 forming the beginning of the tail that anchors the INTS10–INTS13–INTS14 module^34^ to the core of Integrator. The ‘sting’ at the end of the tail is formed by the INTS13–INTS14 dimer. The sting projects towards the upstream DNA and it is positioned by INTS10, the INTS14 vWA domain and the INTS13 cleavage module binding motif (CMBM)^34^ (Figs. 1 and 2).

**Extended Data Fig. 5:**
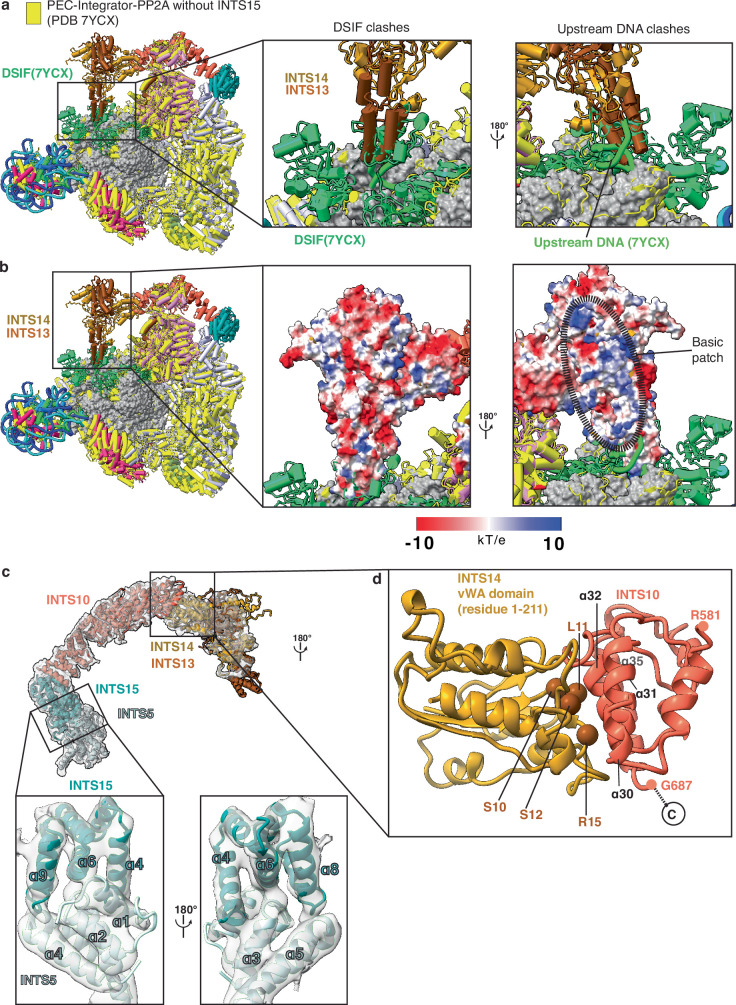
Details of tail-module interfaces and competition with DSIF and upstream DNA. **a**,**b**, Superposition of the pre-termination complex structure and a structure of PEC–Integrator–PP2A without the tail module (PDB 7YCX) shows that the INTS13–INTS14 sting would clash with the upstream DNA and the DSIF DNA clamp. A close-up view of the INTS13–INTS14 sting shown as surface and coloured by electrostatic potential^64^ is presented in **b**. **c**, Cryo-EM density around the tail module. A close-up view showing the INTS5 and INTS15 interface is shown. **d**, Interaction between INTS10 and INTS14. The INTS14 residues essential for this interface^34^ are shown as spheres and labelled.

The subunit INTS15 is a helical protein that contacts the N-terminal region of the Integrator core subunit INTS5. Specifically, the INTS15 N-terminal helices α4 and α6 and a loop between α8 and α9 bind to the INTS5 N-terminal helices α1–α5 (Fig. 2a and Extended Data Fig. 5c). The INTS5 N-terminal region was unresolved in the previous structures of Integrator^1,2^ but is seen here to bind to INTS15. The interface between INTS5 and INTS15 orients the C-terminal helices α17, α20 and α22 of INTS15 to bind to the first two helices of INTS10 and dictate the trajectory of the tail module around the cleavage module (Fig. 2b). This is the only interface between INTS15 and the INTS10–INTS13–INTS14 module and accounts for their physical interaction^23^ (Extended Data Fig. 1a). The lack of the new subunit INTS15 in previous studies explains why the INTS10–INTS13–INTS14 module was not resolved in previous Integrator structures^1,2,44^.

The subunit INTS10 is also a helical protein that runs along the N-terminal HEAT repeats of INTS4 (ref. ^2^) and contacts the interacting C-terminal domain 2 (CTD2)^25^ of INTS9 and INTS11 in the cleavage module (Figs. 1 and 2). The INTS10 C-terminal region between helices α30 and α35 binds to the N-terminal vWA domain^34^ of INTS14, consistent with previously reported bioinformatic and biochemical analyses^34,35^ (Figs. 1 and 2 and Extended Data Fig. 5d). Therefore, INTS10 forms a bridge from INTS15 and helps to position the sting of the tail module. Careful examination of our cryo-EM density map revealed density for the INTS13 residues 655–689 bound to the cleavage module (Fig. 2a and Extended Data Fig. 3g). A similar density was observed previously but could not be modelled^1,2,44^. The INTS13 residues 655–689 correspond to the highly conserved CMBM that is required for interaction between the cleavage module and the INTS10–INTS13–INTS14 module^34^. The CMBM is composed of two α-helices followed by a flexible loop. Whereas the first helix binds to the composite surface formed by the INTS9 and INTS11 CTD1 domains^25^, the second helix and the flexible loop of the CMBM (residues 665–685) form an extended interface with the INTS11 metallo-β-lactamase domain (Fig. 2a). This interface forms a second anchor of the tail module that limits its mobility and helps to position the sting towards the upstream DNA. Furthermore, the linker that connects the CMBM to the INTS13 sting cross-links extensively with INTS9 and INTS11 (Extended Data Fig. 4d), suggesting that it might also help to fix the location of the sting. Mutations in the CMBM are linked to developmental defects in humans^46^, highlighting the importance of this interface for the biological function of Integrator during development.

## Role of INTS13–INTS14 in Pol II termination

Superposition of our pre-termination structure onto the structure of PEC–Integrator–PP2A without the tail module^44^ shows that the sting of the tail module would clash with upstream DNA and the DSIF DNA clamp (Extended Data Fig. 5a,b). Thus, this module would compete with and displace the DSIF DNA clamp and the upstream DNA, which would facilitate the release of the nucleic acid from the Pol II cleft^37^ and is therefore predicted to facilitate Pol II termination. The surface charge potential of the INTS13–INTS14 dimer that forms the sting is largely negative, except for a small basic patch that may contact the upstream DNA^34^ (Extended Data Fig. 5b). Both the DSIF DNA clamp and the upstream DNA are mobile in the pre-termination structure (Fig. 1b), consistent with the proposed role of the sting in facilitating Pol II termination, and contrary to a role in DNA binding. The cryo-EM density for the tail module shows that it is dynamic and might accommodate both the DSIF DNA clamp and the upstream DNA in some conformational states. Furthermore, the location of the tail module might enable the INTS13–INTS14 dimer that is positioned close to upstream DNA to bind to upstream transcription factors, to recruit Integrator to specific gene loci for termination^33^. The importance of the tail module is highlighted by the genetic diseases that are associated with mutations in its subunits^38,46^.

## Integrator nuclease degrades nascent RNA

Whereas opening of the DSIF clamp by the tail module may facilitate the release of nucleic acids from the Pol II cleft, termination generally requires the unravelling of the DNA–RNA hybrid in the Pol II cleft by factors such as the exonuclease XRN2, the translocase Sen1 or the endonuclease CPSF73, all of which act on the exiting nascent RNA^47,48^. To investigate whether the Integrator nuclease INTS11 also exhibits such a function, we performed RNA cleavage assays using our previously reported protocol^1^. To monitor the length of the RNA bound inside Pol II after Integrator activity, RNA was fluorescently labelled at its 3′ end (Fig. 1c,d (top) and Methods). We found that Integrator containing wild-type INTS11 or the less active INTS11(E203Q) mutant cleaved PEC-associated RNA about 20 nt from the Pol II active site, consistent with previous reports^1,44^. As negative controls, the catalytically inactive variant INTS11(D72K/H73A) was unable to cleave the 35-nt PEC-associated RNA, and the wild-type Integrator could not cleave a 17-nt PEC-associated RNA that is completely protected inside Pol II (Fig. 1c,d and Supplementary Fig. 2).

However, the wild-type INTS11 degraded the Pol II-bound RNA to oligonucleotides that are clearly shorter than the 10-nt RNA that is present in the DNA–RNA hybrid (Fig. 1c,d and Supplementary Fig. 2). These results indicate that the INTS11 nuclease does not just catalyse a single cut in the exiting nascent RNA, but that it can also degrade nascent RNA further. This observation is consistent with a function of Integrator in unravelling the DNA–RNA hybrid to trigger Pol II termination.

## Structure of the post-termination complex

**Extended Data Fig. 6:**
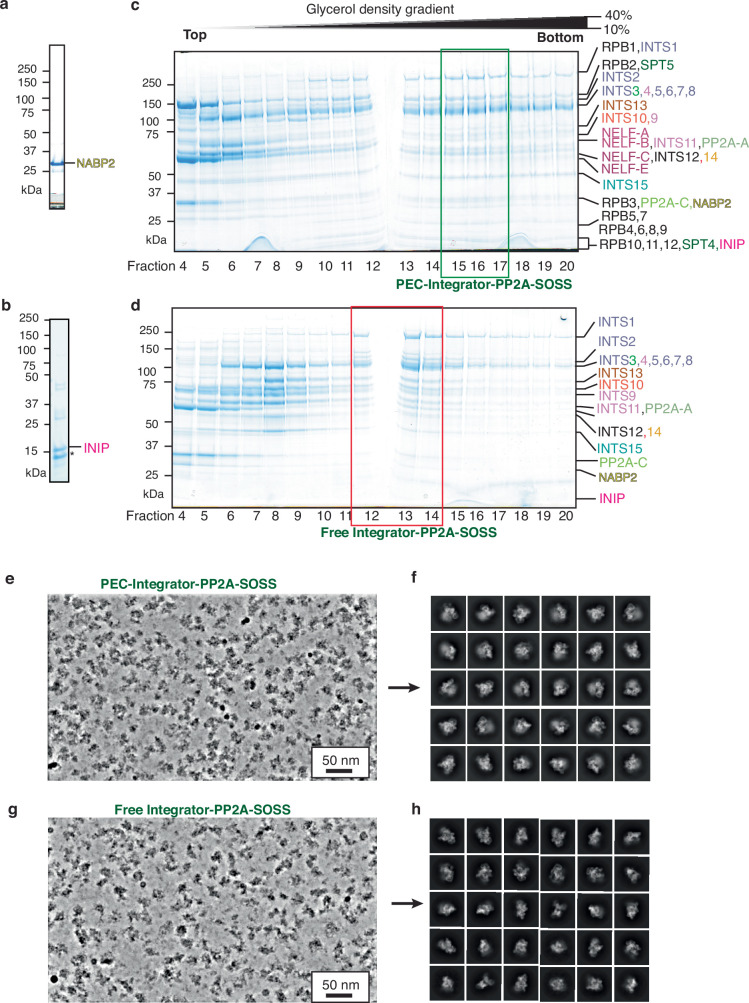
Biochemical reconstitution of the PEC–Integrator–PP2A–SOSS and free Integrator–PP2A–SOSS complexes and cryo-EM images. **a**, SDS–PAGE of purified NABP2. **b**, SDS–PAGE of purified INIP. INIP degradation band is shown by *. **c**,**d**, SDS–PAGE of fractions from glycerol-gradient ultracentrifugation analyses of the PEC–Integrator–PP2A–SOSS complex and the free Integrator–PP2A–SOSS complex. Proteins were identified by mass spectrometry. **e**,**f**, Representative denoised micrograph of fraction 16 from the glycerol-gradient analysis of the PEC–Integrator–PP2A–SOSS complex and two-dimensional class averages of selected good particles. **g**,**h**, A representative denoised micrograph of fraction 13 from the glycerol-gradient analysis of the free Integrator–PP2A–SOSS complex and two-dimensional class averages of selected good particles.

**Extended Data Fig. 7:**
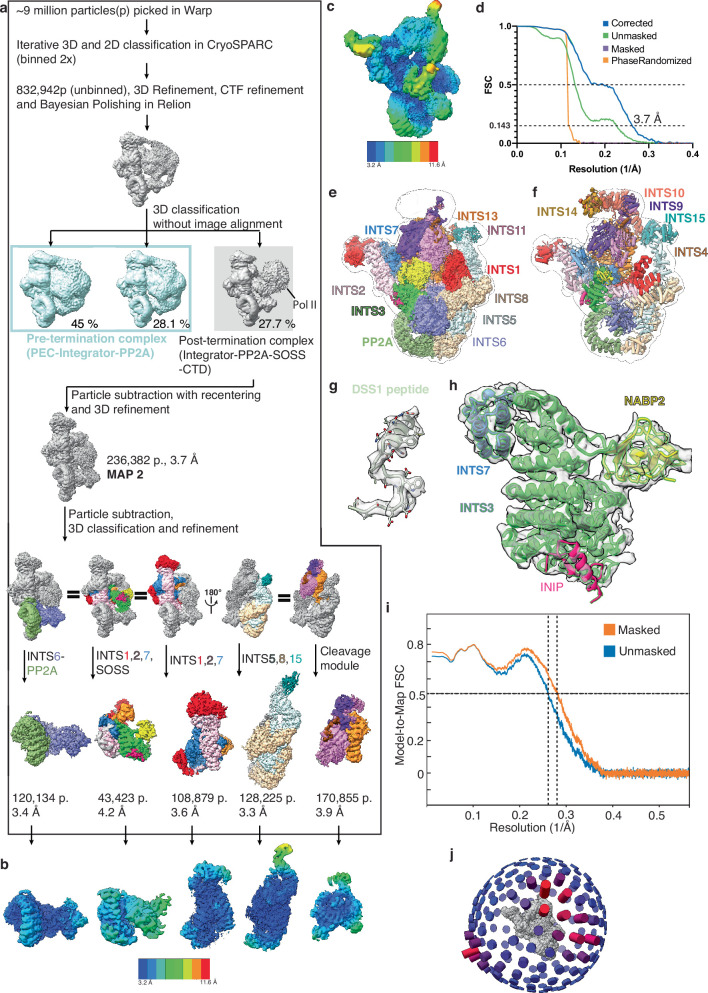
Cryo-EM analysis and modelling of the post-termination complex. **a**, Flow chart showing data-processing steps. **b**, Local resolution of focused refinement maps. A key showing resolution values of the various coloured regions is provided. **c**, Overall cryo-EM reconstruction of the post-termination complex coloured according to local resolution. **d**, FSC plot for the overall cryo-EM reconstruction. The gold-standard FSC at 0.143 is indicated. **e**, The focused refinement maps used for modelling fitted into a low-pass-filtered version of the overall reconstruction shown by the black outline. **f**, The overall structure fitted into the low-pass-filtered version of the overall reconstruction. **g**, Cryo-EM density for DSS1. **h**, Cryo-EM densities around the INTS7 N terminus, INTS3, NABP2 and INIP. **i**, Model-to-map FSC plot. **j**, Angular distribution plot of the overall reconstruction.

**Extended Data Fig. 8:**
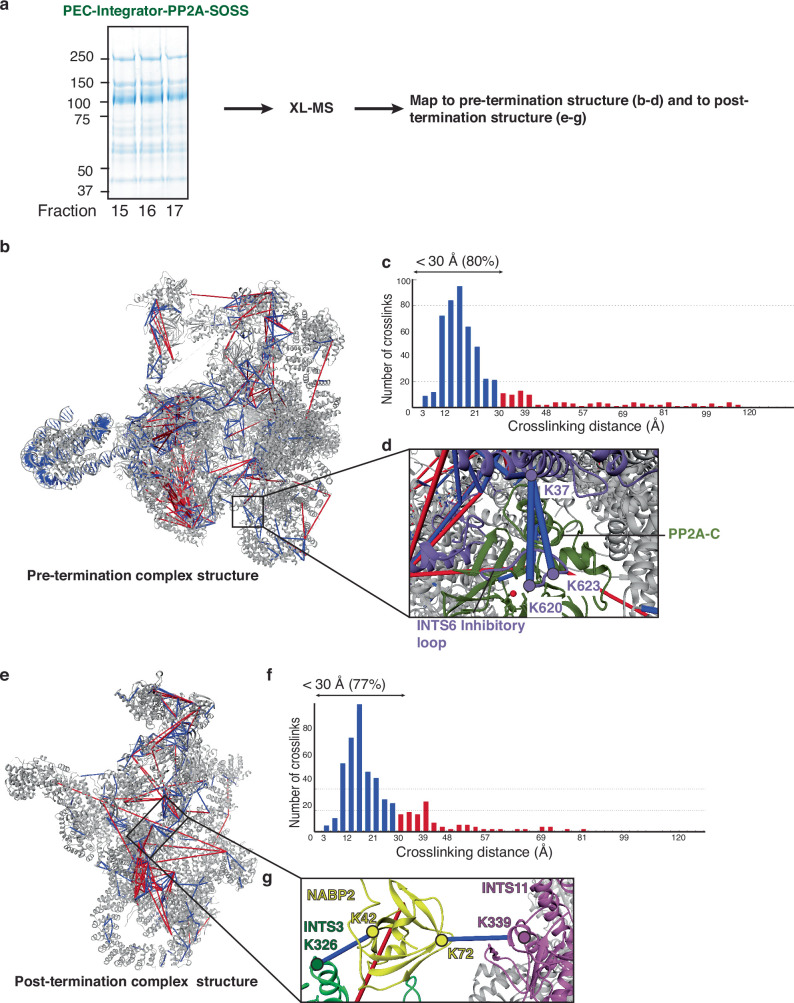
XL-MS analysis of the PEC–Integrator–PP2A–SOSS complex. **a**, SDS–PAGE of the peak fractions of the PEC–Integrator–PP2A–SOSS complex that was used for XL-MS analysis. The full gel is shown in Extended Data Fig. 6. Note that this complex contains a mixed population of the pre-termination complex of PEC–Integrator–PP2A–SOSS and the post-termination complex of Integrator–PP2A–SOSS–CTD according to our cryo-EM analysis shown in Extended Data Fig. 7a. **b**, Mapping of the cross-links obtained from the PEC–Integrator–PP2A–SOSS complex shown in **a** on the pre-termination complex structure using Xlink Analyzer^71^. For clarity, only cross-links with more than ten cross-linked peptide spectrum matches were included. **c**, Histogram showing the distribution of distances between Cα pairs of cross-linked Lys residues in the pre-termination complex structure. **d**, Close-up view showing cross-links from INTS6 K37 to INTS6 K620 and K623 that supports the location of the INTS6 inhibitory loop. **e**, Mapping of the cross-links obtained from the PEC–Integrator–PP2A–SOSS complex shown in **a** on the post-termination complex structure. **f**, Histogram showing the distribution of distances between Cα pairs of cross-linked Lys residues in the post-termination complex structure. **g**, Close-up view showing BS3 cross-links from NABP2 to INTS3 and INTS11.

To investigate how NABP2 and INIP interact with Integrator at Pol II pause sites, we reconstituted a PEC–Integrator–PP2A complex as previously described^1^ using the 15-subunit Integrator, and also included recombinant human NABP2 and INIP (Methods and Extended Data Fig. 6a–d). Glycerol-gradient ultracentrifugation and mass-spectrometric analyses showed that these factors bound to the PEC–Integrator–PP2A complex to form a PEC–Integrator–PP2A–SOSS complex (Extended Data Fig. 6c). Cryo-EM analysis of this complex led to two reconstructions (Extended Data Figs. 6e,f and 7a). The first reconstruction is similar to the structure of the pre-termination complex and the published PEC–Integrator–PP2A complex^1^, and was not analysed further. The second reconstruction corresponds to an Integrator–PP2A–SOSS–CTD post-termination complex and contains cryo-EM density for part of the Pol II CTD and Integrator–PP2A, including the N-terminal region of the flexible subunit INTS3 and the SOSS factors (Extended Data Fig. 7a). We refined the post-termination complex reconstruction to 3.7 Å and used focused classification to improve the resolution of various parts, allowing us to build an atomic model (Extended Data Fig. 7b–j, Extended Data Table 1, Supplementary Video 2 and Methods). XL-MS analysis of the PEC–Integrator–PP2A–SOSS complex produced cross-links that agree with the presence of a pre- and a post-termination complex in this sample (Extended Data Fig. 8 and Supplementary Table 2).

**Fig. 3:**
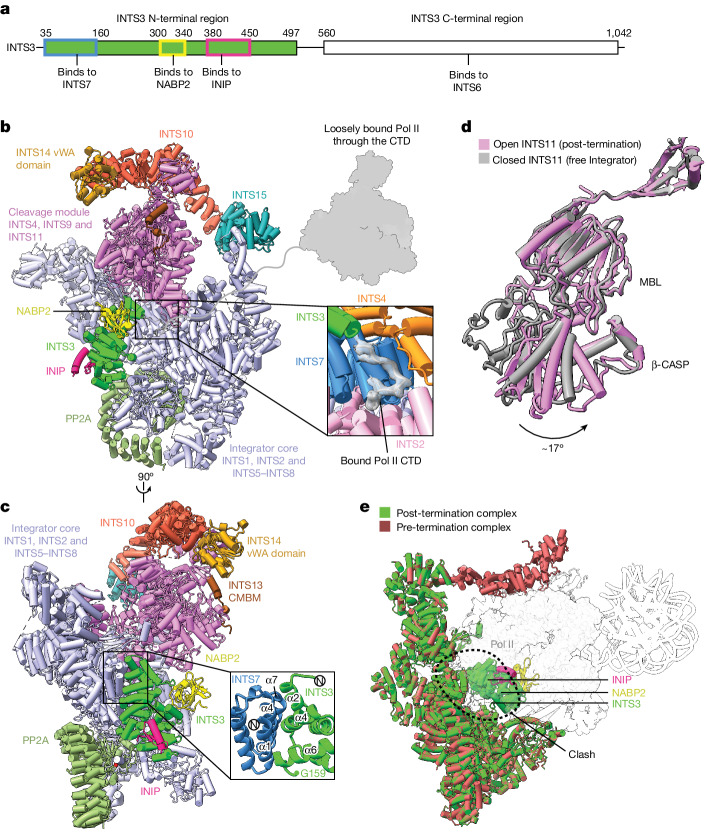
Cryo-EM structure of the post-termination complex. **a**, Organization of the Integrator subunit INTS3. Regions bound by INTS6, INTS7, NABP2 and INIP are shown. **b**, Cryo-EM structure of the post-termination complex. A cartoon of the loosely attached Pol II and the cryo-EM density for the stably bound Pol II CTD peptide are shown. **c**, Alternative view of the complex. A magnified view of the INTS3–INTS7 interface is shown. **d**, Open conformation of INTS11 in the post-termination structure compared with its closed conformation in the free Integrator–PP2A structure. **e**, Superposition of the structures of the pre-termination complex and the post-termination complex shows that INTS3, NABP2 and INIP would clash with Pol II.

The post-termination structure is reminiscent of Integrator in its open conformation, with bound Pol II CTD peptides, and INTS11 is in the open conformation, as observed in the PEC-bound Integrator–PP2A structures^1,44^ (Fig. 3a–d). The N-terminal region of INTS3 (residues 35–500) was resolved bound to the INTS7 N-terminal region. In particular, INTS3 α2, α4 and α6 bind to INTS7 α1, α4 and α7 (Fig. 3c, Extended Data Fig. 7h and Supplementary Video 2). The SOSS factors NABP2 and INIP bind to INTS3, as observed in the reported crystal structure^31^. The SOSS factor NABP2 cross-links to INTS11 in the cleavage module, thereby trapping this post-termination intermediate; this explains why it was not observed in previous studies that lacked NABP2^1,44^ (Extended Data Fig. 8g). Comparison with our pre-termination structure shows that the presence of INTS3 and SOSS factors is incompatible with Pol II binding (Fig. 3e). This suggests that INTS3 binds to the open conformation of Integrator and blocks the rebinding of Pol II to Integrator after termination.

## Complete Integrator–PP2A structure

**Extended Data Fig. 9:**
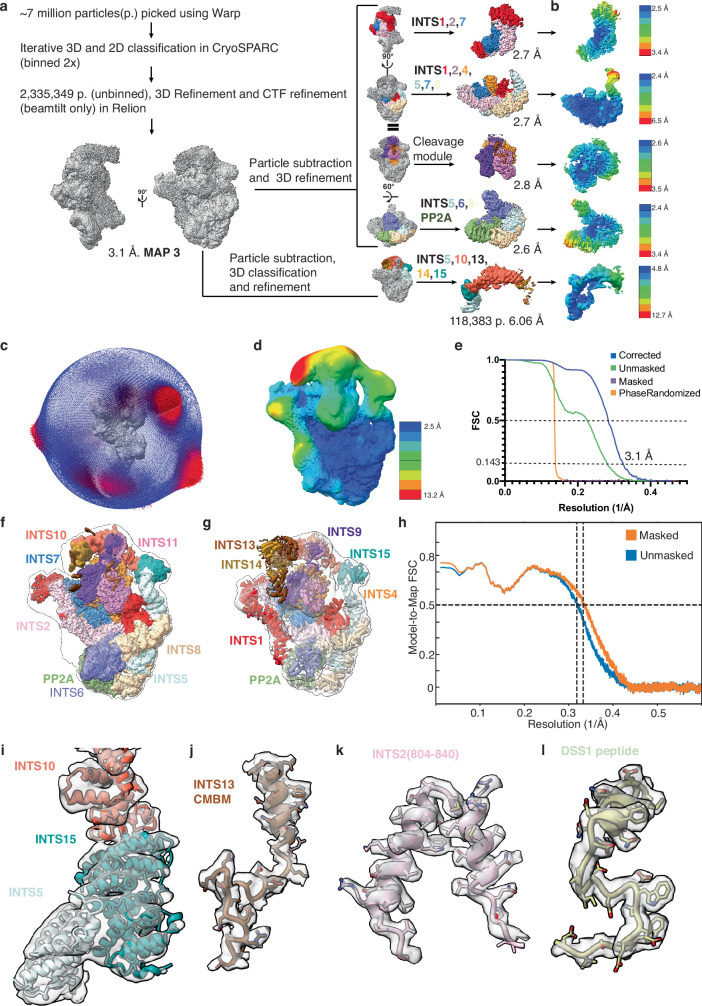
Cryo-EM analysis and modelling of the free Integrator–PP2A complex. **a**, Flow chart showing the cryo-EM data-processing procedure. **b**, Local resolution of focused refined maps. Keys showing resolution values of the various coloured regions are provided. **c**, Angular distribution plot of the overall reconstruction. **d**, Overall cryo-EM reconstruction of the free Integrator–PP2A coloured according to local resolution. **e**, FSC plot for the overall cryo-EM reconstruction. The gold-standard FSC at 0.143 is indicated. **f**, The combined focused refinement maps used for modelling fitted into a low-pass-filtered version of the overall reconstruction that is indicated by the black outline. **g**, The overall structure fitted into the low-pass-filtered version of the overall reconstruction. **h**, Model-to-map FSC plot. **i**, Cryo-EM density for the INTS5 N terminus, INTS15 and the INTS10 N terminus. **j**, Cryo-EM density for the INTS13 CMBM. **k**, Cryo-EM density for INTS2(804-840). **l**, Cryo-EM density for DSS1.

In the structure of the post-termination complex, we resolved the INTS3 N-terminal region, which was not observed in the previous structure of free Integrator–PP2A that lacked NABP2 and INIP (ref. ^2^). We therefore asked whether the INTS3 N-terminal region would bind to INTS7 in the absence of the PEC. We mixed Integrator–PP2A, NABP2 and INIP and confirmed complex formation by glycerol-gradient ultracentrifugation and mass-spectrometric analyses (Methods and Extended Data Fig. 6d). We determined a cryo-EM structure of the complex at a resolution of 3.1 Å (Extended Data Figs. 6g,h and 9a–e). We docked the structure of Integrator–PP2A taken from our pre-termination structure into the cryo-EM density map. The model was adjusted using ISOLDE^40^ and refined in real space using PHENIX^42^ (Extended Data Fig. 9f–l, Extended Data Table 1 and Methods).

**Fig. 4:**
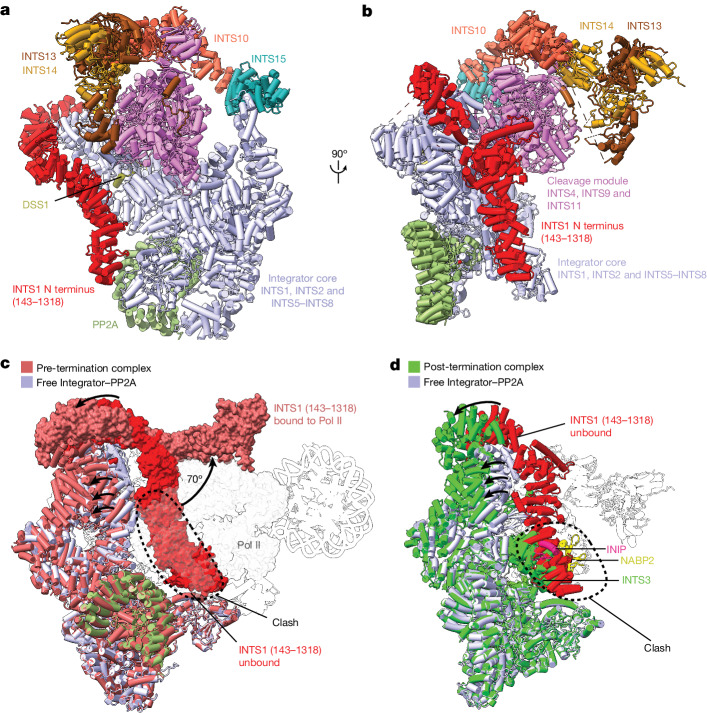
Cryo-EM structure of the free Integrator–PP2A complex. **a**, Cryo-EM structure of the free Integrator–PP2A complex. The locations of the INTS1 N-terminal region and the co-purified *Trichoplusia ni* DSS1 are shown. **b**, Alternative view of the complex. **c**, Superposition of the structures of the pre-termination complex and the free Integrator–PP2A complex shows that the INTS1 N-terminal region would clash with Pol II. **d**, Superposition of the structures of the free Integrator–PP2A complex and the post-termination complex shows that INTS3, NABP2 and INIP would clash with INTS1. Numbers in brackets indicate residues.

The obtained structure reveals Integrator in a closed state, resembling the reported structure of the free Integrator–PP2A complex^1,2^. The higher resolution enabled us to improve the previous model of the free Integrator–PP2A complex and to include the tail module and the N-terminal region of INTS1 (residues 143–1318) (Fig. 4a,b, Extended Data Fig. 9f,g and Supplementary Video 3). The Integrator subunits INTS3 and INTS12 and the SOSS factors were not present in this structure, although mass spectrometry confirmed their presence in the cryo-EM sample.

## Post-termination role of INTS3

In the free Integrator–PP2A structure, the INTS1 N-terminal region (residues 143–906) that binds to Pol II in the PEC–Integrator–PP2A structure^1^ is moved inwards by a rotation of around 70°, and approaches INTS2 and INTS6 (Fig. 4c and Extended Data Fig. 9f,g). Superposition on our pre-termination structure shows that the location of the INTS1 N-terminal region is incompatible with Pol II binding (Fig. 4c and Supplementary Video 4). The INTS1 N-terminal region in the free Integrator–PP2A structure also occludes the binding of INTS3, which instead binds to the open Integrator conformation in the post-termination structure (Fig. 4d).

These findings support our model of INTS3 and SOSS factors in Integrator-dependent Pol II termination. SOSS factors bind to INTS3 in the free Integrator and are present at Pol II pause sites because of interactions between the flexible C-terminal regions of INTS3 and INTS6 (refs. ^13,29,30^). Binding to the PEC stabilizes the open conformation of Integrator and exposes the INTS3-binding surface of INTS7, which is concurrently blocked by a stably bound Pol II in the pre-termination complex. RNA cleavage and destabilization of the Pol II elongation complex by the cleavage and tail modules of Integrator allow INTS3 to transiently bind to the INTS7 surface that is located between Integrator and Pol II, to prevent Pol II from reassociating with Integrator. In the free Integrator, NABP2 can bind to single-stranded DNA to recruit Integrator to certain loci, as recently suggested^32^, but it is unlikely to do the same in the post-termination state. In summary, our complete structure of the free Integrator–PP2A complex suggests that the N-terminal region of INTS1 has a role in dislodging INTS3 from INTS7 in the post-termination complex (Supplementary Video 4). This would allow Integrator to adopt the closed conformation that can open to bind to the PEC.

## INTS6 blocks the PP2A active site

**Fig. 5:**
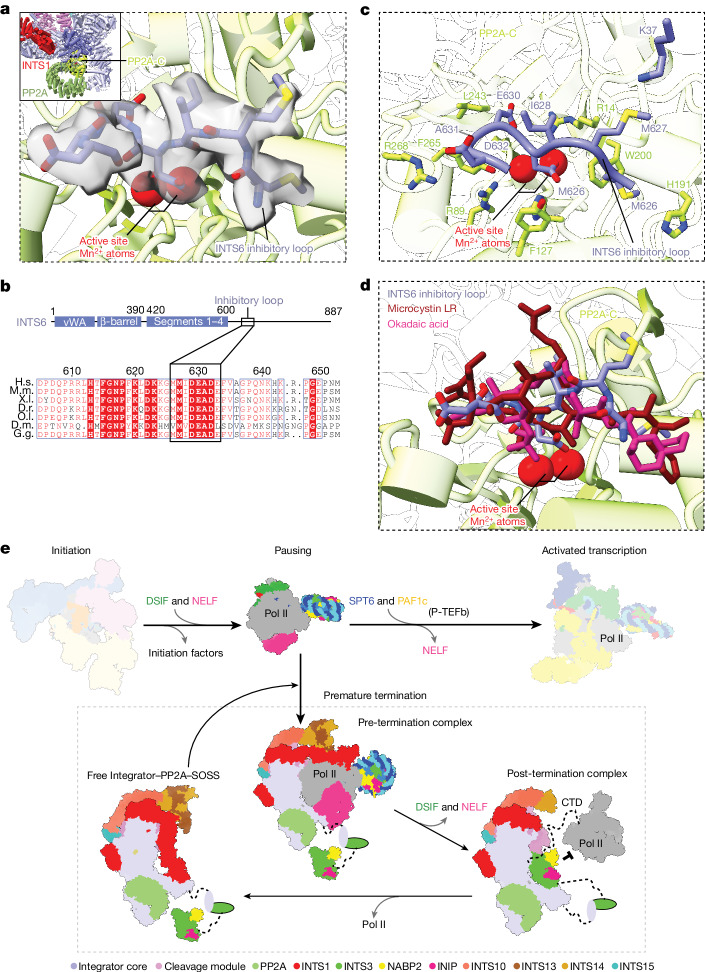
The Integrator subunit INTS6 regulates PP2A, and a model of the Integrator-dependent Pol II termination cycle. **a**, Magnified view showing the cryo-EM density of the INTS6 inhibitory loop bound in front of the PP2A-C active site in the free Integrator–PP2A structure. **b**, Top, organization of the Integrator subunit INTS6, showing the location of the Inhibitory loop in the disordered C-terminal region. Bottom, protein sequence alignment showing the conservation of the inhibitory loop across species. H.s., *Homo sapiens*; M.m., *Mus musculus*; X.l., *Xenopus laevis*; D.r., *Danio rerio*; O.l., *Oryzias latipes*; D.m., *Drosophila melanogaster*; G.g., *Gallus gallus*. **c**, Magnified view showing details of the INTS6 inhibitory loop and PP2A-C interactions. Interacting residues are shown as sticks and labelled. INTS6 K37, which cross-links with INTS6 K620 and K623 that are located just outside the inhibitory loop, is shown. **d**, Comparison of the INTS6 inhibitory loop and the PP2A-C inhibitors okadaic acid and microcystin-LR. **e**, Model for Integrator-dependent Pol II termination. Pol II pauses after transcription initiation when bound by DSIF and NELF. P-TEFb kinase activity and the binding of SPT6 and PAF1c can release paused Pol II into activated transcription. Alternatively, the Integrator–PP2A complex binds to paused Pol II and dephosphorylates the Pol II CTD, then degrades the exiting nascent RNA transcript and releases the bound DNA to terminate Pol II. The Integrator subunit INTS3 (and associated NABP2 and INIP) then binds to INTS7 in the open conformation to facilitate the removal of Pol II from the Integrator–PP2A complex. The INTS1 N-terminal region displaces INTS3 and the SOSS factors and Integrator can return to the closed conformation, ready to bind to the PEC for another termination cycle.

Inspection of our cryo-EM density map of the free Integrator–PP2A complex revealed a density for a peptide that occupies the active centre of PP2A-C (Fig. 5a). We modelled and assigned this density to INTS6 residues 626–633, hereafter called the INTS6 inhibitory loop (Fig. 5). The INTS6 acidic residues D629 and E630 within a conserved DEAD motif of the INTS6 inhibitory loop mimic phosphoserine and phosphothreonine residues and bind to the PP2A-C active site to block substrate binding (Fig. 5b,c). The INTS6 inhibitory loop forms additional contacts with residues around the PP2A-C active site, including R89, H191, W200, F265 and R268 (Fig. 5c). These residues are also bound by the known PP2A-C inhibitors okadaic acid and microcystin-LR (ref. ^49^), showing that there is a marked similarity between the INTS6 inhibitory loop and these toxins (Fig. 5d). The INTS6 inhibitory loop is highly conserved (Fig. 5b).

We also observed cryo-EM density for the INTS6 inhibitory loop in our pre-termination complex (Extended Data Fig. 3e). Our XL-MS data show that INTS6 K37, which is located near the PP2A-C active site, cross-links with INTS6 K620 and K623, which are located just a few residues from the start of the INTS6 inhibitory loop (Fig. 5c and Extended Data Figs. 4c and 8d). These indicate that the INTS6 inhibitory loop is not displaced by the unphosphorylated Pol II CTD, contrary to a previous suggestion^44^. Conversely, purified Integrator–PP2A dephosphorylates the Pol II CTD^2^, suggesting that the phosphorylated CTD can displace the inhibitory loop in vitro. Jointly, these results suggest a conserved inhibitory function of INTS6 on PP2A that ensures that the phosphatase is inactive in the free Integrator–PP2A complex. Because the INTS11 nuclease is also inactive in the free Integrator^1,44^, both enzymatic activities are likely to be switched off unless a functional higher-order complex with the PEC is formed.

## Discussion

Integrator-dependent premature termination is emerging as an integral way to regulate Pol II transcription^20,21,50^, but our understanding of how Integrator terminates Pol II is limited. Here we present three cryo-EM structures of the complete Integrator: one bound to the PEC in the pre-termination state; one bound to Pol II in the post-termination state; and one of the complete, free complex in an inactive state. The structure of the pre-termination complex reveals the previously unresolved Integrator tail module, which is shaped like the tail of a scorpion, including a sting. The tail module binds to the Integrator core and the cleavage module to position its sting and displace the DSIF DNA clamp^37^, and to interfere with upstream DNA. The post-termination structure shows that the INTS3 N-terminal region binds to INTS7 in the open conformation of Integrator and prevents the rebinding of Pol II to Integrator after termination. Finally, the structure of the free Integrator–PP2A complex reveals that, in the absence of the PEC, the complex adopts an inactive state, with the INTS1 N-terminal region interfering with the binding of INTS3 and INTS6 occupying the PP2A-C active site.

Together with biochemical data and published work, our structures suggest a three-step model for Integrator-dependent Pol II termination. First, the inactive Integrator–PP2A complex undergoes conformational rearrangements to open and bind to the PEC, which positions the PP2A phosphatase to enable dephosphorylation of the Pol II CTD, counteracting the kinase activity of P-TEFb. Second, the Integrator tail module displaces the DSIF DNA clamp and upstream DNA, and the Integrator endonuclease INTS11 cleaves the nascent RNA and degrades it further to unravel the DNA–RNA hybrid and trigger a collapse of the transcription bubble, which releases DNA from Pol II. We speculate that RNA degradation and DNA release are coupled, because the tail and cleavage modules are connected by the INTS13 CMBM. Third, Pol II dissociates from Integrator and its rebinding is prevented by INTS3 and the associated SOSS factors, which bind to the INTS7 surface and sterically prevent the formation of a complex with Pol II. At the end, Integrator adopts a closed, inactive conformation, ready for another termination cycle (Fig. 5e and Supplementary Video 4).

This model for Integrator-dependent Pol II termination shows conceptual similarity with current models of Pol II termination by other factors. In particular, the torpedo model applied at the end of protein-coding genes involves the 5′−3′ exonuclease XRN2, which degrades nascent RNA from its 5′ end and unravels the DNA–RNA hybrid, leading to a collapse of the transcription bubble and Pol II termination^47,48,51,52^. The Integrator endonuclease INTS11 is closely related to the endonuclease CPSF73, which has also been suggested to serve as a torpedo nuclease for the termination of replication-dependent histones^53^. Given that Integrator-dependent promoter-proximal termination and the termination of noncoding Pol II transcripts require NELF-dependent pausing of Pol II (refs. ^1,20,21,50,54^), a putative 5′−3′ exonuclease activity of INTS11 might be sufficient to unravel the DNA–RNA hybrid bound by Pol II. How INTS11 reaches the RNA bound inside the Pol II cleft after the first cleavage is unclear. We speculate that INTS11 remains bound to the 5′ end of the Pol II-associated cleaved RNA, because the INTS11 active-centre groove accommodates around 5 nt of the exiting nascent RNA before the site of cleavage^1,44^. This might allow Integrator to translocate towards the RNA 3′ end as it degrades the RNA 5′ end and push Pol II forward in the process. Consistent with our model, Pol II termination at the Integrator-dependent noncoding loci in metazoans is independent of XRN2 (ref. ^51^). It however remains to be seen under which circumstances termination is achieved by Integrator alone and when Integrator might cooperate with XRN2 to achieve termination.

## Methods

### Molecular cloning and protein expression and purification

The constructs used for expressing Integrator were described previously^1^ with some modification. In brief, INTS1 and INTS15 cDNA sequences were codon-optimized for protein expression in *T. ni* (Hi5 insect cells) and the cDNAs were purchased from Integrated DNA Technologies (IDT). Owing to its size, the cDNA for INTS1 was divided into three fragments for synthesis. The codon-optimized INTS1 cDNA fragments were cloned into vector 438-C (Addgene 55220) and combined with INTS12, which was cloned in vector 438-A (Addgene 55218), to create the INTS1–INTS12 construct. The cDNA for INTS15 was cloned into vector 438-C. Expression constructs for the INTS2–INTS3–INTS5–INTS6–INTS7–INTS8 subcomplex, the cleavage module, the INTS10–INTS13–INTS14 module and the PP2A complex were described previously^1^.

Full-length human NABP2 (Q9BQ15-1) and INIP (Q9NRY2-1) cDNAs were codon-optimized for Hi5 insect cells, purchased from IDT and individually cloned into vector 438-B (Addgene 55219) by ligation-independent cloning^55^.

Baculoviruses for protein expression in insect cells were generated in SF9 and SF21 cells (Thermo Fisher Scientific) using a previously described protocol^56^. We expressed the eight-subunit Integrator core by co-infecting Hi5 cells (Thermo Fisher Scientific) with two baculoviruses, one expressing the INTS2–INTS3–INTS5–INTS6–INTS7–INTS8 subcomplex with an N-terminal 6×His-MBP tag on INTS5 and the other containing the INTS1–INTS12 construct. We expressed the tail module (INTS10–INTS13–INTS14–INTS15) by co-infecting Hi5 cells with baculoviruses containing 6×His-MBP–INTS15 and the INTS10–INTS13–INTS14 module. The PP2A complex and the Integrator cleavage module were expressed as previously described^1^. NABP2 and INIP were also expressed in Hi5 cells using baculoviruses generated from their respective constructs.

The eight-subunit Integrator core, the cleavage module (and its mutants) and PP2A were purified using the published protocols^1^. The tail module was purified essentially as described for the INTS10–INTS13–INTS14 module^1^ except that we used amylose instead of Ni affinity.

To prepare NABP2, Hi5 insect cells expressing the protein were collected by centrifugation at 238*g* for 30 min in a high-speed centrifuge (Beckman Coulter) operated at 4 °C. The supernatant was discarded and the cell pellet from a 1.2-l culture was resuspended in 80 ml lysis buffer (50 mM Tris-HCl pH 7.5, 800 mM NaCl, 20 mM imidazole, 10% (v/v) glycerol, 2 mM EDTA and 5 mM DTT). Cells were lysed by sonication with 30% amplitude for 2 min with a 0.4-s pulse on and a 0.6-s pulse off using a Branson digital sonifier. The lysate was spun at 87,207*g* in a high-speed centrifuge at 4 °C for 1 h and filtered with a 0.8-μm syringe filter to remove cell debris. The clarified lysate was applied to a pre-equilibrated 5-ml HisTrap HP column (Cytiva) at a flow rate of 1.5 ml per min and the column was washed with 100 ml lysis buffer. The bound protein was eluted from the column using a gradient from 0–100% over 18 column volumes of an Ni elution buffer (50 mM Tris-HCl pH 7.5, 800 mM NaCl, 500 mM imidazole, 10% (v/v) glycerol, 2 mM EDTA and 5 mM DTT). The fractions containing NABP2 were combined and treated with 5 mg 6×His-TEV protease and lambda protein phosphatase, and dialysed overnight against 800 ml low-salt buffer (50 mM Tris-HCl pH 7.5, 150 mM NaCl, 20 mM imidazole, 10% (v/v) glycerol, 2 mM EDTA and 5 mM DTT) at 4 °C in a 7-kDa molecular weight cut-off (MWCO) SnakeSkin dialysis tubing (Thermo Fisher Scientific). The digested sample was applied to a 5-ml HisTrap HP column to remove uncleaved protein and TEV protease. The flow-through fraction was applied to a pre-equilibrated 5-ml HiTrap SP HP column. NABP2 was recovered in the flow-through, concentrated in an Amicon 15-ml centrifugal filter (10 kDa MWCO) (Millipore) to around 1.0 ml and applied to a Superdex 75 10/300 GL column (Cytiva) equilibrated in 50 mM HEPES pH 7.5, 300 mM NaCl, 10% (v/v) glycerol and 2 mM TCEP. Peak fractions were analysed by SDS–PAGE, and the fractions that contain pure NABP2 were concentrated, aliquoted, flash-frozen and stored at −80 °C.

To prepare INIP, Hi5 cells were collected, lysed, filtered and clarified using the protocol for NABP2. Cells were resuspended in a low-salt lysis buffer (20 mM MES pH 6.1, 150 mM NaCl, 20 mM imidazole and 2 mM DTT). Cleared lysate was applied to a pre-equilibrated 5-ml HisTrap HP column (Cytiva) at a flow rate of 1.5 ml per min and the column was washed with 100 ml low-salt lysis buffer. Elution was performed using Ni elution buffer (20 mM MES pH 6.1, 150 mM NaCl, 500 mM imidazole and 2 mM DTT), and peak fractions were analysed by SDS–PAGE. Fractions containing INIP were combined with lambda phosphatase and dialysed overnight at 4 °C in SnakeSkin dialysis tubing (3.5 kDa MWCO) (Thermo Fisher Scientific) against 800 ml low-salt lysis buffer. After dialysis, the protein was applied to a pre-equilibrated 5-ml HiTrap SP HP cation-exchange column. INIP-containing fractions after ion exchange were pooled and concentrated in an Amicon 15-ml centrifugal filter (3 kDa MWCO) (Millipore) to around 1.0 ml and applied to a Superdex 75 10/300 GL column (Cytiva) equilibrated in 50 mM HEPES pH 7.5, 300 mM NaCl, 10% (v/v) glycerol and 2 mM TCEP. Peak fractions were concentrated, aliquoted, flash-frozen in liquid nitrogen and stored at −80 °C.

Preparation of mammalian Pol II (ref. ^57^) human DSIF (ref. ^37^), NELF (ref. ^5^) and human histones^58^ was done as described in the corresponding references.

### Nucleosome preparation

DNA fragments for nucleosome reconstitution were generated by PCR as described previously^59^. In brief, nucleosome DNA was amplified from a vector containing the 145-bp Widom 601 sequence and a 40-bp run-up sequence upstream of the Widom 601. A 50-ml PCR was performed using the following primers: forward: 5′-GCAGTCCAGTTACGCTGGAGTC-3′ and reverse: 5′ATCAGAATCCCGGTGCCG −3′. The sequence of the PCR product is 5′-GCAGTCCAGTTACGCTGGAGTCTGAGGCTCGTCCTGAATGATATGCGGCCTCACGAAGCGTAGCATCACTGTCTTGTGTTTGGTGTGTCTGGGTGGTGGCCGATATCGATGTATATATCTGACACGTGCCTGGAGACTAGGGAGTAATCCCCTTGGCGGTTAAAACGCGGGGGACAGCGCGTACGTGCGTTTAAGCGGTGCTAGAGCTGTCTACGACCAATTGAGCGGCCTCGGCACCGGGATTCTGAT-3′. PCR product purification, TspRI digestion, octamer formation, nucleosome reconstitution and purification of nucleosome with a PrepCell system were performed as previously described^59^. The concentration of the reconstituted and purified nucleosome was determined using the sum of the molar extinction coefficients of DNA and octamer at 280 nm and the absorbance of the nucleosome at this wavelength. Super-helical locations were assigned on the basis of previous publications (see references in ref. ^59^).

### RNA extension assays

We performed in vitro RNA extension assays to identify the NELF-dependent Pol II pause site in the nucleosome. A 5′ Cy5-labelled RNA (5′-Cy5/rUrUrArUrCrArCrUrGrUrC-3′) that anneals at the TspRI-generated overhang in the run-up to the nucleosome was used to load Pol II onto the nucleosomal substrate for RNA extension. Assays were performed in a volume of 10 µl in a final buffer containing 100 mM NaCl, 20 mM Na-HEPES, pH 7.4, 5 mM MgCl_2_, 1 mM DTT and 4% glycerol. Depending on the reaction, RNA (480 nM) was incubated with either DNA substrate or nucleosomal substrate (240 nM) for 10 min on ice. *Sus scrofa* Pol II (300 nM) was added to the reaction and incubated for 10 min on ice. DSIF (600 nM), TFIIS (180 nM) and buffer were added to the samples. Transcription was initiated by adding 0.5 mM each of GTP, CTP, UTP and ATP or 3′-dATP with or without NELF (600 nM). After a 60-min incubation at 30 °C, 5 µl transcription reaction was quenched with 5 µl of a 2× Stop buffer (6.4 M urea, 50 mM EDTA, pH 8.0 and 2× TBE buffer). Quenched samples were treated with 1.6 units of proteinase K (NEB) for 30 min at 37 °C and denatured for 10 min at 95 °C before the fluorescent RNA was separated using denaturing PAGE (8 M urea, 1× TBE buffer, 12% acrylamide:bis-acrylamide 19:1 gel; run for 42 min at 300 V in 0.5× TBE buffer). RNA products were visualized by their Cy5 label in a Typhoon 9500 FLA imager.

### Reconstitution of complexes for cryo-EM and XL-MS analyses

#### Pre-termination complex

The RNA extension assay showed that the presence of NELF impaired Pol II transcription to the nucleosomal substrate as compared with Pol II–DSIF alone. We thus used a two-step procedure to form the pre-termination complex for cryo-EM and XL-MS analyses. First, a transcribed Pol II–DSIF–Nuc complex was reconstituted in a buffer containing 100 mM NaCl, 20 mM HEPES, pH 7.4, 5 mM MgCl_2_, 1 mM DTT and 4% (v/v) glycerol. The 5′ Cy5-labelled RNA (3.2 µM) and the nucleosomal substrate (1.6 µM) were mixed and incubated for 5 min on ice. *S. scrofa* Pol II (2 µM) was added to the reaction and incubated for another 5 min on ice. We added DSIF (6 µM) and 3′-dATP (1 mM) and the mixture was incubated for 10 min at 30 °C. Transcription was started by adding TFIIS (1.2 µM), CTP, GTP and UTP (each 1 mM), and it proceeded for 60 min at 30 °C in a final volume of 50 µl. In parallel, we mixed 3.8 µM each of the eight-subunit Integrator core, cleavage module, tail module and PP2A in a final volume of 80 µl and incubated on ice to form the integrator–PP2A complex. We used an INTS11(E203Q) mutant that has reduced catalytic activity in all complexes formed for cryo-EM and XL-MS analyses.

In the second step, we added the preformed Integrator–PP2A complex and NELF (2 µM) to the transcribed Pol II–DSIF–Nuc complex in a final buffer comprising 156 mM NaCl, 28 mM HEPES, pH 7.4, 5 mM MgCl_2_, 1 mM DTT and 4% glycerol in a final volume of 200 µl. We incubated the mixture for 30 min at 30 °C. The assembled pre-termination complex was purified using a 4-ml 10–40% glycerol gradient as previously described^1^. Samples removed after each step were analysed using denaturing PAGE.

#### PEC–Integrator–PP2A–SOSS and free Integrator–PP2A–SOSS complexes

The PEC–Integrator–PP2A–SOSS complex from which we obtained the post-termination structure was formed essentially as described for the PEC–Integrator–PP2A complex using the published DNA scaffolds^1,5^ and a variant of the HIV TAR RNA that does not form a secondary structure. The RNA has the following sequence, 5′-/6-FAM/rUrUrArArGrGrArArUrUrArArGrUrCrGrUrGrCrGrUrCrUrArArUrArAr CrCrGrGrArGrArGrGrGrArArCrCrCrArCrU-3′. We pre-incubated 3.8 µM each of the eight-subunit Integrator core, Integrator cleavage module, tail module, PP2A, NABP2 and INIP in a final volume of 80 µl on ice to form the Integrator–PP2A–SOSS complex. We formed the PEC using 0.6 µM of Pol II, 1.2 µM each of nucleic acids and 1.8 µM of DSIF and NELF. The preformed Integrator–PP2A–SOSS complex was added to the PEC in a final volume of 163 µl. We incubated the mixture for 30 min at 30 °C and applied it to a 10–40% glycerol.

The free Integrator–PP2A–SOSS complex was formed by mixing 3.8 µM of each of the Integrator–PP2A subcomplexes with 3.8 µM of NABP2 and INIP on ice for 60 min. The complex was purified using a 10–40% glycerol gradient.

### Cryo-EM sample preparation

Peak fractions from the glycerol-gradient analyses of the pre-termination, PEC–Integrator–PP2A–SOSS and free Integrator–PP2A–SOSS complexes were separately cross-linked using 0.2% (v/v) glutaraldehyde for 10 min on ice. The cross-linking reaction was quenched using 100 mM Tris-HCl (pH 8) for 10 min on ice. The cross-linked cryo-EM samples were dialysed for 4–6 h against a buffer containing 20 mM HEPES pH 7.4, 150 mM NaCl, 1% (v/v) glycerol, 3 mM MgCl_2_, 1 mM DTT and 0.01% (w/v) CHAPS using a 20 kDA MWCO Slide-A-Lyzer MINI Dialysis Unit (Thermo Fisher Scientific). A 2.6–2.8-µm-thin carbon film was floated on the dialysed cryo-EM samples and incubated for 5–15 min depending on the concentration of the sample before cross-linking. The floated carbon film was transferred onto a Quantifoil R3.5/1 copper mesh 200 grid and instantly blotted for 2 s with blot force 5 before being vitrified in liquid ethane using a Vitrobot Mark IV (Thermo Fisher Scientific). The vitrobot was operated at 4 °C and 95–100% humidity.

### Cryo-EM data collection and processing

All cryo-EM data were acquired at a nominal magnification of 81,000×, corresponding to a calibrated pixel size of 1.05 Å per pixel, using a K3 direct electron detector (Gatan) on a Titan Krios transmission electron microscope (Thermo Fisher Scientific) operated at 300 kV. Images were collected in EFTEM mode using a Quantum LS energy filter (Gatan) with a slit width of 20 eV. A defocus range of −0.5 to −2.0 μm was applied during data collection and images were recorded in electron counting mode. The SerialEM software^60^ was used for automated data acquisition. Motion correction of collected movies, dose weighting, constrast transfer function (CTF) estimation and particle picking were performed using Warp^61^.

For the pre-termination complex sample, we collected 59,687 micrographs with a dose rate of 14.86 e^−^ per pixel per s for 3 s, resulting in a total dose of 40.44 e^−^ per Å^2^ that was fractionated into 50 movie frames. Micrographs with bad CTF fits in Warp were excluded from further processing. We extracted 9,107,060 picked particles with a box size of 500 pixels and binned 2× to a pixel size of 2.1 Å per pixel using RELION 3.1 (ref. ^62^). These particles were subjected to heterogenous refinement in CryoSPARC (ref. ^63^) using initial models generated from our previous data^1^. The selected good particles that had cryo-EM density for the PEC and the Integrator–PP2A complex were further sorted using two-dimensional (2D) classification in CryoSPARC. We identified 1.3 million good particles with this procedure, which were re-extracted in RELION 3.1 (ref. ^62^) without binning. We performed one round of three-dimensional (3D) classification in RELION to eliminate Integrator–PP2A particles that have only a weak Pol II density, resulting in 278,693 particles. This set of particles were CTF refined and polished in RELION 3.1 to obtain a 3.8-Å reconstruction encompassing the PEC and Integrator–PP2A. We applied soft masks around various parts of this map and performed signal subtraction, 3D classification and refinement in RELION 3.1. This produced good focused refined maps better than 3.5 Å that aided model building. A subset of 80,717 particles was obtained from focused classification with a mask around the PEC. We reverted the signal for these particles and performed global 3D refinement to obtain the overall reconstruction for the pre-termination complex at a resolution of 4.1 Å (map 1). The density for the nucleosome was very weak, showing that it is highly dynamic in this complex.

For the PEC–Integrator–PP2A–SOSS sample that led to the post-termination structure of the Integrator–PP2A–SOSS–CTD complex, 52,976 micrographs were acquired. Each micrograph was acquired with a 2.84-s exposure at a dose rate of 15.50 e^−^ per pixel per s, resulting in a total dose of 39.93 e^−^ per Å^2^ that was divided into 40 movie frames. We excluded micrographs with a bad CTF fit and extracted 9,165,848 particles in RELION 3.1 (ref. ^62^) using a 480-pixel box size. The extracted particles were binned to a pixel size of 2.1 Å per pixel to speed up initial sorting. Bad particles were removed using iterative 3D and 2D classification in CryoSPARC as described above, resulting in 832,842 good particles. This set of particles was taken through Bayesian polishing, CTF refinement and 3D refinement procedures in RELION 3.1. We identified three main classes when we applied 3D classification without image alignment. The first two classes are similar to the published PEC–Integrator–PP2A complex^1^. The third class of 236,382 particles led to the reconstruction of the post-termination complex. We applied a soft mask around this class to subtract out the weak Pol II density that could not be resolved owing to flexibility. Three-dimensional refinement of the signal-subtracted particles led to a 3.7-Å overall reconstruction for the post-termination complex (map 2). We improved the resolution of local regions of the map using signal subtraction, 3D classification and refinement.

We collected 47,268 micrographs using a grid prepared from the free Integrator–PP2A–SOSS complex. The images were collected with 2.82-s exposures with a dose rate of 15.83 e^−^ per pixel per s and a total dose of 40.49 e^−^ per Å^2^ that was split into 40 movie frames. We extracted 7,014,615 particles with a box size of 480 pixels that we binned to 2.1 Å per pixel after preprocessing the data in Warp. We used 3D and 2D classification in CryoSPARC to remove junk and broken particles, resulting in 2,335,349 good particles that we re-extracted without binning and subjected to CTF and 3D refinement procedures in RELION 3.1. We obtained a 3.1-Å reconstruction using the above steps. We performed signal subtraction with recentring of the particles followed by 3D refinement to obtain cryo-EM maps better than 2.9 Å for various regions of Integrator–PP2A. For the tail module, we performed 3D classification on the signal-subtracted particles to identify a subset set of 118,383 particles that refined to 6.1 Å. Further classification of these particles did not improve the resolution of the tail module.

### Model building and refinement

To build a model for the pre-termination complex, we first fitted the model of PEC–Integrator–PP2A (PDB ID: 7PKS) into map 1 using ChimeraX (ref. ^64^) and adjusted the fit using focused refined maps. Manual adjustments to the model were made in Coot (ref. ^65^) after initial rounds of ISOLDE flexible fitting^40^. We determined the sequence register of nucleic acids bound in the Pol II cleft using the 3.2-Å PEC focused refinement map. Following this register, we built the DNA–RNA hybrid and extended the downstream DNA. The downstream nucleosome was modelled by rigid-body-docking a structure of the nucleosome (PDB ID: 7OHC) into the low-pass-filtered version of map 1 using the sequence register from the downstream DNA.

For the tail module, we rigid-body-docked AlphaFold2 (ref. ^39^) models for INTS10 and INTS15 into the focused refined map of this module and adjusted them in Coot and ISOLDE. We predicted the interface between the C terminus of INTS10 and INTS14 using Colabfold (ref. ^41^). The predicted model was fitted in the focused refined map and adjusted using ISOLDE. The available crystal structure of INTS13–INTS14 (PDB ID: 6SN1)^34^ was aligned on our model of INTS10–INTS14 to derive the correct orientation of the INTS13–INTS14 sting. This naturally placed the INTS13–INTS14 sting inside the low-pass-filtered map 1. The INTS13 CMBM, INTS6 inhibitory loop and DSS1 models were copied from the structure of the free Integrator–PP2A and manually adjusted using Coot. Various parts of the model were refined against respective focused refined maps using the phenix.real_space_refine tool in the PHENIX package^42,66^. The final model was refined against map 1 with reference model restraints to account for regions with weak cryo-EM density in the consensus refinement.

We built a model for the post-termination complex by copying the Integrator–PP2A structure from the pre-termination complex structure above. The model was first fitted into the overall map of the post-termination complex (map 2) and adjusted in Coot and ISOLDE using the focused refined maps to fit residue side chains. INTS1 residues 1–866 and the INTS13–INTS14 sting were removed from the model because cryo-EM density for these regions was lacking. The crystal structure of the SOSS complex containing INTS3(1–501), NABP2 and INIP (PDB ID: 4OWW)^31^ was docked into the focused refinement map around INTS3 and the SOSS factors. Regions lacking cryo-EM density were removed and the model was further adjusted using ISOLDE. A combined model for the post-termination complex was created in map 2 and subjected to real-space refinement in the PHENIX package^42,66^.

The model for free Integrator–PP2A was built by first docking a structure of Integrator–PP2A that was copied from the pre-termination complex structure into map 3. ISOLDE was used to adjust the model to fit the map. The higher-resolution focused refined maps were used to fit side chains. Our XL-MS data suggested that the INTS6 inhibitory loop binds in front of the PP2A-C active site. We used Colabfold to predict possible interfaces between the flexible INTS6 C terminus and PP2A-C. We found one predicted model that perfectly matched our cryo-EM density and used it to model the INTS6 inhibitory loop. The interface between INTS11 and the INTS13 CMBM was also at first predicted using Colabfold and subsequently adjusted using ISOLDE and Coot. To identify the DSS1 peptide in our cryo-EM density and build a model for it, we subjected the 2.7-Å focused refined map around INTS1–INTS2–INTS7 to sequence-free de novo modelling using ModelAngelo (ref. ^67^). The software identified and modelled the conserved C-terminal part of DSS1 into our cryo-EM density. The modelled part of the *T. ni* DSS1 corresponds to residues 35–60 of the human orthologue. We did not assign a sequence numbering because there is no database with annotated *T. ni* DSS1. An AlphaFold2 model for the N terminus of INTS1 (residues 143-905) was rigid-body-docked into the overall map to complete the model. The final model was refined in real space using PHENIX^42,66^.

The following regions were built as backbone traces where applicable because side-chain information was absent in our cryo-EM density maps. INTS1(143–906), INTS10, INTS13(11-564), INTS14 and INTS15.

### Integrator RNA degradation assay

RNA cleavage and degradation activity of Integrator was tested using the RNA cleavage assay preciously described^1^. In brief, a completely complementary template and non-template DNA and a single-stranded RNA that anneals to the template DNA were purchased from IDT. The nucleic acids have the following sequences: template DNA, 5′-GCTTTATTGAGGCTTAAGCAGTGGGTTCCAGGTACTAGTGTACAGCTATCGTAAGCTATCGTAGGCAAGGTCCACTGACT/3Bio/-3′; non-template DNA, 5′-AGTCAGTGGACCTTGCCTACGATAGCTTACGATAGCTGTACACTAGTACCTGGAACCCACTGCTTAAGCCTCAATAAAGC-3′; and RNA, 5′-rArGrUrCrGrUrGrCrGrUrCrUrArArUrArArCrCrGrGrArGrArGrGrGrAr ArCrCrCrArCrU/3Cy5Sp/-3′. Note that the RNA has a 3′ Cy5 fluorescent label for visualizing the Integrator cleavage products.

A 6× PEC master mix was prepared using 450 nM Pol II, 90 nM of each nucleic acid and 900 nM each of DSIF and NELF in a final volume of 40 µl. Aliquots of the PEC master mix (6.7 µl) were treated individually with 150 nM preformed Integrator–PP2A complexes containing wild-type INTS11, an INTS11(E203Q) mutant that has reduced enzymatic activity or an INTS11(D72K/H73A) mutant that is catalytically dead, or were not treated with Integrator. Reactions were performed in 40-µl final volumes in buffer R (20 mM HEPES pH 7.4, 150 mM NaCl, 10% (v/v) glycerol, 3 mM MgCl_2_, 1 mM DTT, 1 U μl^−1^ RNAsin plus (Promega)). For the free Integrator control, the preformed wild-type Integrator was mixed with the annealed DNA–RNA scaffold. All reactions were incubated at 30 °C for 1 h, quenched and analysed on a denaturing PAGE as described^1^.

The above protocol was used for the time-course RNA cleavage and degradation by wild-type Integrator shown in Fig. 1d and Supplementary Fig. 2f,g, except for the following. For a more efficient PEC formation, the PEC scaffold that contains a mismatch bubble^5^ was used instead of the completely complementary scaffold. We used a 25-nt or 17-nt RNA that has a 3′ Cy5 label to show that Integrator cannot act on RNA that is covered inside Pol II. The RNA sequences are shown in Fig. 1d and Supplementary Fig. 2f,g.

### Chemical cross-linking coupled with mass spectrometry

Chemical cross-linking was performed using the peak fractions from the pre-termination complex and the PEC–Integrator–PP2A–SOSS glycerol-gradient ultracentrifugation. For each complex, we ran two gradients using the protocol described above and pooled the peak fractions. This was required to get sufficient material for XL-MS. The pooled peak fractions were cross-linked with 3 mM BS3 for 30 min at 30 °C and quenched with 100 mM Tris-HCl (pH 8.0). Mass spectrometry was performed as previously described^1^, except for the following. For PEC–INT–PP2A–SOSS BS3-cross-linked peptides were pre-fractionated by size exclusion or by C18 basic pH reverse-phase chromatography (bRP). For PEC–Nuc–INT–PP2A, only bRP pre-fractionation was performed. Both samples were measured in triplicate in a Thermo Orbitrap Exploris mass spectrometer without FAIMS installed (Thermo Fisher Scientific). BS3-mediated protein-protein cross-links were identified using pLink 2.3.11 (http://pfind.org/software/pLink/).

### Visualization

Protein sequence alignments were made with Multalign (ref. ^68^) and visualized with ESPRIPT 3.0 (ref. ^69^). Structural figures were made with UCSF Chimera (ref. ^70^) and ChimeraX (ref. ^64^). The Supplementary Videos were made in ChimeraX. Please note that Supplementary Video 4 was made by interpolating between the various conformations of proteins in our structures and the trajectory of protein domains may not necessarily reflect intermediate conformations.

### Reporting summary

Further information on research design is available in the Nature Portfolio Reporting Summary linked to this article.

## Online content

Any methods, additional references, Nature Portfolio reporting summaries, source data, extended data, supplementary information, acknowledgements, peer review information; details of author contributions and competing interests; and statements of data and code availability are available at 10.1038/s41586-024-07269-4.

### Supplementary information

Supplementary Information This file contains Supplementary Figures 1 & 2

Reporting Summary

Supplementary Table 1 Cross-linking mass-spectrometry analysis of the PEC–Nucleosome–Integrator–PP2A pre-termination complex

Supplementary Table 2 Cross-linking mass-spectrometry analysis of the PEC–Integrator–PP2A–SOSS complex

Supplementary Video 1 Cryo-EM structure of the pre-termination complex

Supplementary Video 2 Cryo-EM structure of the post-termination complex

Supplementary Video 3 Cryo-EM structure of the free Integrator–PP2A complex

Supplementary Video 4 The Integrator termination cycle

## Figures and Tables

**Extended Data Table 1:**
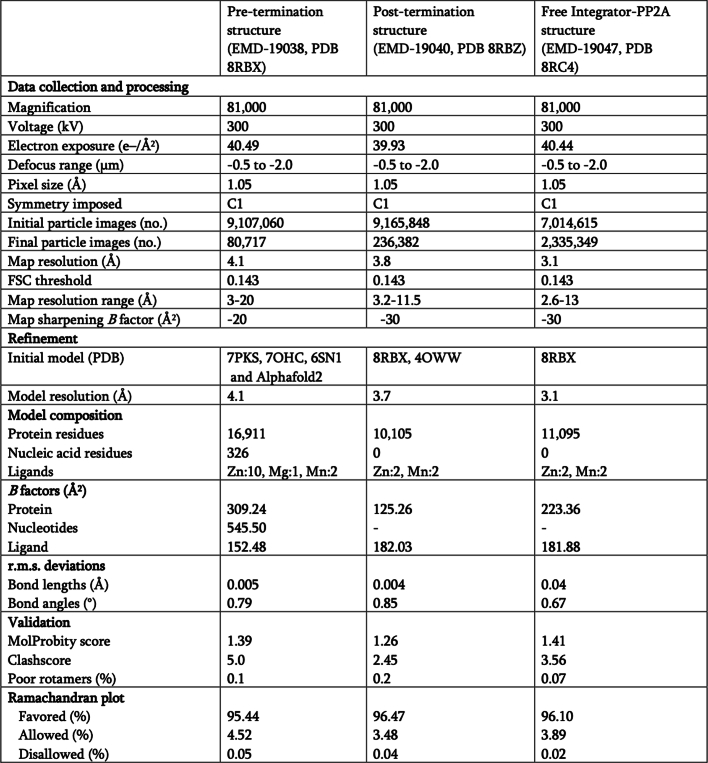
Cryo-EM data collection, refinement and validation statistics

## Data Availability

The cryo-EM reconstructions and final models have been deposited in the Electron Microscopy Data Bank under accession codes EMD-19038 (pre-termination complex), EMD-19040 (post-termination complex) and EMD-19047 (free Integrator–PP2A), and in the PDB under accession codes 8RBX (pre-termination complex), 8RBZ (post-termination complex) and 8RC4 (free Integrator–PP2A). The following models were used as input for structural building: PDB IDs 7PKS, 7OHC, 6SN1 and 4OWW, and an AlphaFold2 model.
