# Supplementary material for: Structural basis of Integrator-dependent RNA polymerase II termination
Source: Nature. 2024 Apr 3;629(8010):219–27. doi: 10.1038/s41586-024-07269-4 (PMC11062913; doi:10.1038/s41586-024-07269-4)
Supplement: Supplementary file 1 — This file contains Supplementary Figures 1 & 2 [file 41586_2024_7269_MOESM1_ESM.pdf]

nature portfolio
https://doi.org/10.1038/s41586-024-07269-4

Supplementary information

Structural basis of Integrator-dependent RNA polymerase II termination

In the format provided by the authors and unedited

Structural basis of Integrator-dependent RNA polymerase II termination

Isaac Fianu[1*], Moritz Ochmann[1], James L. Walshe[1], Olexandr Dybkov[2], Joseph Neos Cruz[1], Henning Urlaub[2,3,4], and Patrick Cramer[1*]

Supplementary Figure S1
Title: Conservation of DSS1 and its interaction with INTS7.

Supplementary Figure S2
Title: RNA cleavage and degradation assays.

Supplementary Table 1
Title: Crosslinking mass spectrometry analysis of the PEC-Nucloeosome-Integrator-PP2A pre-termination complex.

Supplementary Table 2
Title: Crosslinking mass spectrometry analysis of the PEC-Integrator-PP2A-SOSS pre-termination complex.

Supplementary Video 1
Title: Cryo-EM structure of the pre-termination complex
A video showing the pretermination structure fitted into a lowpass filtered version of the overall cryo-EM reconstruction.

Supplementary Video 2
Title: The post-termination structure
A video showing the post-termination structure. Cryo-EM density for Pol II CTD, NABP2, INIP and INTS3 are shown.

Supplementary Video 3
Title: Cryo-EM structure of free Integrator-PP2A complex
A video showing the free Integrator-PP2A structure fitted into a lowpass filtered version of the overall reconstruction. Cryo-EM density for INTS6 inhibitory loop, DSS1 and INTS7 are shown.

Supplementary Video 4
Title: The Integrator termination cycle
An animation of the Integrator termination cycle shown in Fig. 5e. Integrator opens to bind the PEC and positions its nuclease and PP2A to degrade the nascent RNA and dephosphorylate Pol II CTD respectively. The sting of INTS13-14 opens the DSIF clamp and interferes with upstream DNA to facilitate Pol II termination. INTS3 prevents Pol II rebinding to Integrator. Finally, INTS1 displaces INTS3 in the absence of Pol II.

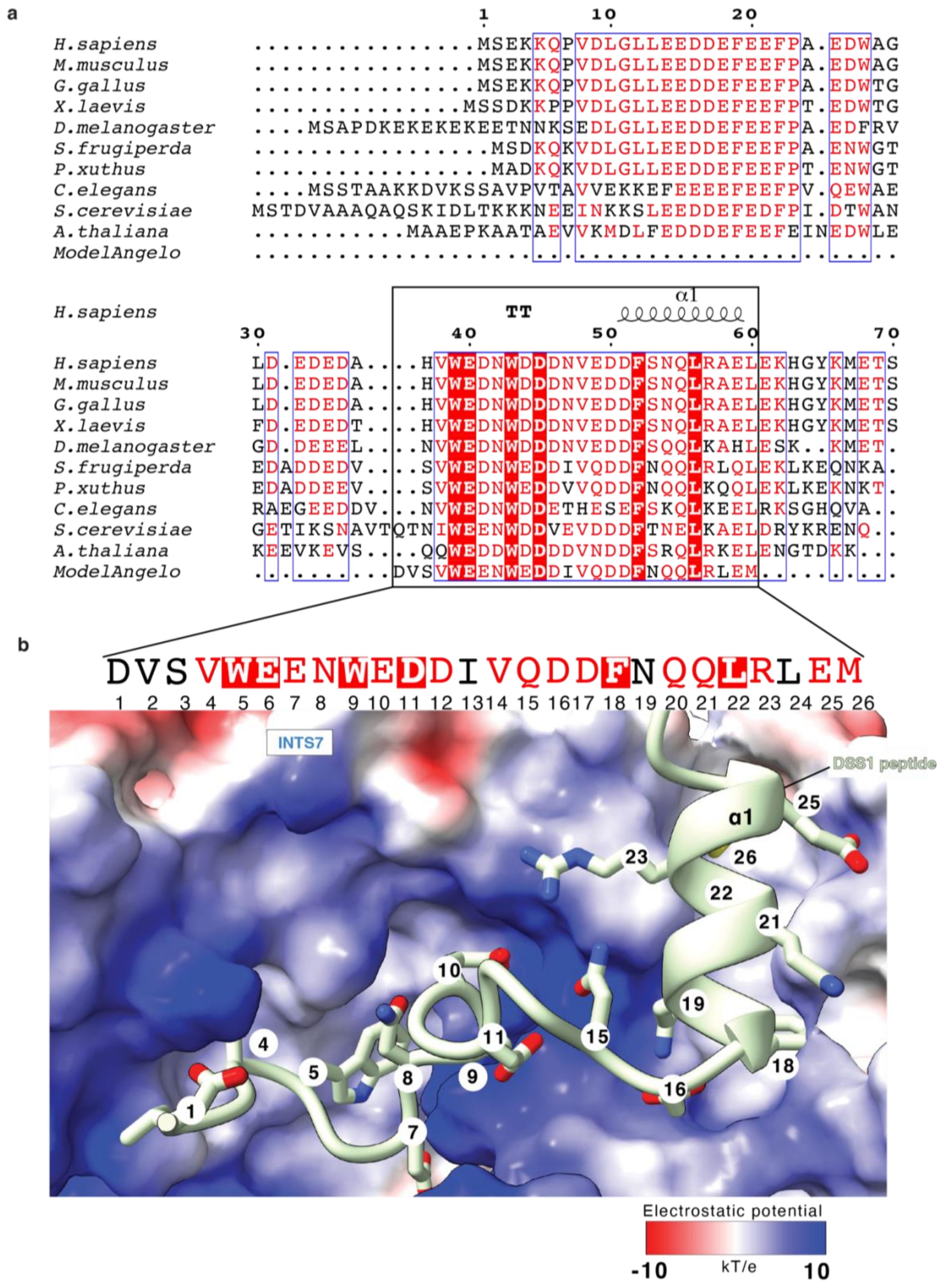

Supplementary Fig. S1| Conservation of DSS1 and its interaction with INTS7.

a. Sequence alignment showing the conservation of DSS1 across species. *ModelAngelo,* represents the sequence obtained from *de novo* modeling using Modelangelo[67]. **b.** The DSS1 peptide is shows as a cartoon bound to INTS7 that is shown as a surface colored according to electrostatic potential. The observed peptide corresponds to the conserved C-terminal region of DSS1. We observed a weak density for the N-terminal half but could not model it. Because there is no annotated database with *T. ni DSS1 protein sequence* (the source organism) we do not provide a sequence number for this peptide. We number the residues 1-26 to show the location of interacting residues on INTS7 surface.

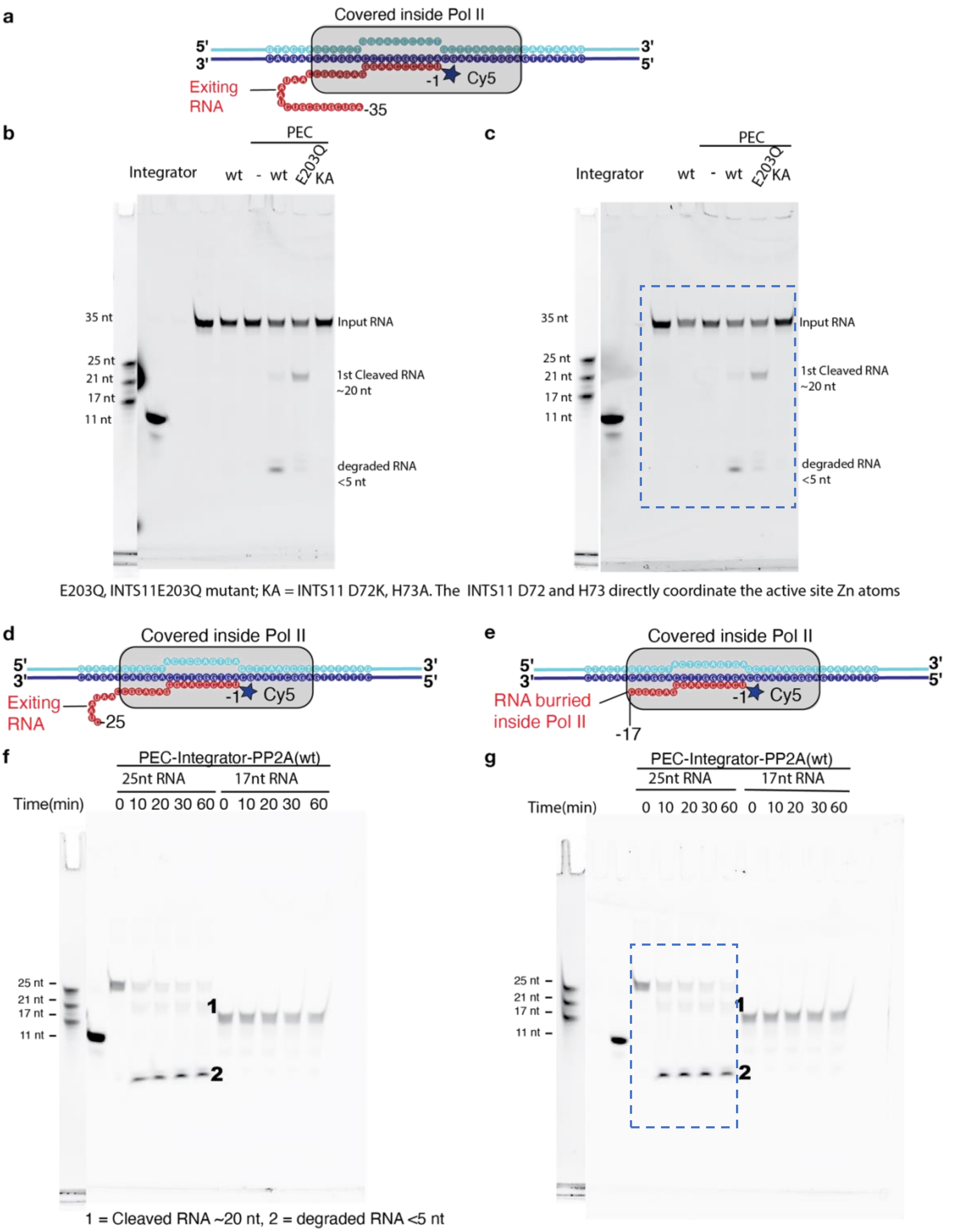

Supplementary Fig. S2| RNA cleavage and degradation assays. a. Nucleic acid scaffold used for assays in panels **b** and **c.** The part of the nucleic acids covered inside Pol II is shown by the rectangle. Please note that the template and non-template strands are complementary. **b-c.** Replicates of the assay performed using the scaffold in panel **a.** The part of the gel shown **Fig. 1c** is indicated by the dashed rectangle.

d-e. Nucleic acid scaffolds used for assays in panels **f** and **g**. Please note these scaffolds contain a mismatch bubble which allows a more stable PEC assembly. **f-g.** Replicates of time dependent RNA degradation assay performed using scaffolds in panels **d** and **e** respectively. Please note Integrator does

not degrade the 17 nt RNA that is completely buried inside Pol II showing it must first cut the exiting nascent RNA at the RNA 5' end outside Pol II. The part of the gel shown **Fig. 1d** is indicated by the dashed rectangle.
