# Supplementary material for: Structural basis of Integrator-dependent RNA polymerase II termination
Source: Nature. 2024 Apr 3;629(8010):219–27. doi: 10.1038/s41586-024-07269-4 (PMC11062913; doi:10.1038/s41586-024-07269-4)
Supplement: Supplementary file 2 — Reporting Summary [file 41586_2024_7269_MOESM2_ESM.pdf]

nature portfolio

Corresponding author(s): Prof. Dr. Patrick Cramer
Dr. Isaac Fianu

Last updated by author(s): Jan 24, 2024

Reporting Summary

Nature Portfolio wishes to improve the reproducibility of the work that we publish. This form provides structure for consistency and transparency in reporting. For further information on Nature Portfolio policies, see our Editorial Policies and the Editorial Policy Checklist.

Statistics

For all statistical analyses, confirm that the following items are present in the figure legend, table legend, main text, or Methods section.

n/a	Confirmed	
☐	☒	The exact sample size (*n*) for each experimental group/condition, given as a discrete number and unit of measurement
☐	☒	A statement on whether measurements were taken from distinct samples or whether the same sample was measured repeatedly
☒	☐	The statistical test(s) used AND whether they are one- or two-sided<br>*Only common tests should be described solely by name; describe more complex techniques in the Methods section.*
☒	☐	A description of all covariates tested
☒	☐	A description of any assumptions or corrections, such as tests of normality and adjustment for multiple comparisons
☐	☒	A full description of the statistical parameters including central tendency (e.g. means) or other basic estimates (e.g. regression coefficient) AND variation (e.g. standard deviation) or associated estimates of uncertainty (e.g. confidence intervals)
☒	☐	For null hypothesis testing, the test statistic (e.g. *F*, *t*, *r*) with confidence intervals, effect sizes, degrees of freedom and *P* value noted<br>*Give P values as exact values whenever suitable.*
☒	☐	For Bayesian analysis, information on the choice of priors and Markov chain Monte Carlo settings
☒	☐	For hierarchical and complex designs, identification of the appropriate level for tests and full reporting of outcomes
☒	☐	Estimates of effect sizes (e.g. Cohen's *d*, Pearson's *r*), indicating how they were calculated

Our web collection on statistics for biologists contains articles on many of the points above.

Software and code

Policy information about availability of computer code

Data collection	SerielEM v4.0
Data analysis	CryoSPARC v3.2.0, Relion3.1, COOT v0.9.4.1, ChimeraX 1.4 and v1.6, UCSF Chimera, PHENIX v1.20.1, WARP v1.0.9, ISOLDE 1.3, ESPRIPT 3.0, Multalign

For manuscripts utilizing custom algorithms or software that are central to the research but not yet described in published literature, software must be made available to editors and reviewers. We strongly encourage code deposition in a community repository (e.g. GitHub). See the Nature Portfolio guidelines for submitting code & software for further information.

Data

Policy information about availability of data

All manuscripts must include a data availability statement. This statement should provide the following information, where applicable:

- Accession codes, unique identifiers, or web links for publicly available datasets
- A description of any restrictions on data availability
- For clinical datasets or third party data, please ensure that the statement adheres to our policy

The cryo-EM reconstructions and final models were deposited with the Electron Microscopy Data Base (EMDB) under accession code EMD-19038 (pre-termination complex structure), EMD-19040 (post- termination complex structure), EMD-19047 (free Integrator-PP2A structure) and with the Protein Data Bank under accession codes 8RBX (pre-termination complex structure), 8RBZ (post- termination complex structure), 8RC4 (free Integrator-PP2A structure).

Input models used are: 7PKS, 7OHC, 6SN1, 4OWW and alphafold2 model.

Research involving human participants, their data, or biological material

Policy information about studies with human participants or human data. See also policy information about sex, gender (identity/presentation), and sexual orientation and race, ethnicity and racism.

Reporting on sex and gender	Not applicable
Reporting on race, ethnicity, or other socially relevant groupings	Not applicable
Population characteristics	Not applicable
Recruitment	Not applicable
Ethics oversight	Not applicable

Note that full information on the approval of the study protocol must also be provided in the manuscript.

Field-specific reporting

Please select the one below that is the best fit for your research. If you are not sure, read the appropriate sections before making your selection.

☒ Life sciences ☐ Behavioural & social sciences ☐ Ecological, evolutionary & environmental sciences

For a reference copy of the document with all sections, see nature.com/documents/nr-reporting-summary-flat.pdf

Life sciences study design

All studies must disclose on these points even when the disclosure is negative.

Sample size	No sample size calculations were made and no statistical methods were used. Cryo-EM data were collected on a grid prepared from one in-vitro reconstituted complex. This is the standard practice in cryo-EM.
Data exclusions	We excluded broken particles and poorly aligned particle classes. This is a routine practice in cryo-EM to exclude such particles because they introduce noise in the reconstruction.
Replication	RNA cleavage assays were performed three times and result were reproducible. Two represent gels are provided in supplementary materials file. Cryo-EM structure determination was not replicated. This is the standard practice.<br>Proteins were prepared using established protocols which normally yield enough material for several reproducible structural and biochemical experiments.
Randomization	Resolution estimation was performed by the gold-standard FSC of two randomly assigned halves of the data set.
Blinding	Not applicable because this is not a clinical studies

Reporting for specific materials, systems and methods

We require information from authors about some types of materials, experimental systems and methods used in many studies. Here, indicate whether each material, system or method listed is relevant to your study. If you are not sure if a list item applies to your research, read the appropriate section before selecting a response.

Materials & experimental systems

n/a	Involved in the study	
☒	☐	Antibodies
☐	☒	Eukaryotic cell lines
☒	☐	Palaeontology and archaeology
☒	☐	Animals and other organisms
☒	☐	Clinical data
☒	☐	Dual use research of concern
☒	☐	Plants

Methods

n/a	Involved in the study	
☒	☐	ChIP-seq
☒	☐	Flow cytometry
☒	☐	MRI-based neuroimaging

Eukaryotic cell lines

Policy information about cell lines and Sex and Gender in Research

Cell line source(s)	Hi5 cells: Expression Systems, T.ni Insect Cells in ESF921 media, Item 94-002F Sf9 cells: ThermoFisher, Catalogue Number 12659017, Sf9 cells in Sf-900TM III SFM Sf21 cells: Expression Systems, Sf21 insect cells in ESF921 medium, Item 94-00
Authentication	provided by supplier (Morphology)
Mycoplasma contamination	Not tested
Commonly misidentified lines (See ICLAC register)	No commonly misidentified cell line was used.
